# Making a Virtue of Necessity: The Use of Wild Edible Plant Species (Also Toxic) in Bread Making in Times of Famine According to Giovanni Targioni Tozzetti (1766)

**DOI:** 10.3390/biology11020285

**Published:** 2022-02-11

**Authors:** Bruno Paura, Piera Di Marzio

**Affiliations:** 1 Department of Agricultural, Environmental and Food Sciences University of Molise, 86100 Campobasso, Italy; 2 Department of Bioscience and Territory, University of Molise, 86090 Pesche, Italy; piera.dimarzio@unimol.it

**Keywords:** Giovanni Targioni Tozzetti, bread, wild edible plants (WEPs), alimurgy, Italy, plant toxicity

## Abstract

**Simple Summary:**

In 1766, while working for the Grand Duchy of Tuscany, Giovanni Targioni Tozzetti listed several plant species that could be used, in times of famine, to increase vegetable mass in bread making. In his text the author cites over 340 vernacular names. We carried out a research work on contemporary and modern bibliographic sources to match them with a binomial name leading to the current nomenclature. In our research we have thus been able to obtain the first “alimurgic flora” drawn up by Targioni Tozzetti himself and made a comparison with our AlimurgITA database of wild edible plants used in Italy. Furthermore, the author identified eight toxic plants that were useful for increasing the mass of bread dough, indicating how to eliminate poisonous substances. We treat them in detail, examining their current and past use, their geographical distribution in Italy, and their possible toxicity. We hope this contribution can stimulate curiosity in the use of wild edible species, even for the ones neglected today because of their unpleasant taste or more complicated use due to their toxicity.

**Abstract:**

In 1766, the agricultural scientist Giovanni Targioni Tozzetti described for the Grand Duchy of Tuscany, the wild and cultivated plant species that could be used, in times of famine, to increase the quantity of flour or vegetable mass in bread making. These wild plants can be defined as wild edible plants (WEPs) or “alimurgic species”, a concept usually traced back to Giovanni Targioni Tozzetti himself. The 342 plant names mentioned in the text are in the Tuscan vernacular, so a research work was done on bibliographic sources from the 1800s in order to match them with their current nomenclature. This process led to an “alimurgic flora” repertoire based on the writing of Targioni Tozzetti; and a comparison with our AlimurgITA database of 1103 wild edible plants used in Italy. It is particularly interesting that in his short treatise, Giovanni Targioni Tozzetti identified eight toxic plants (corresponding to 14 species), indicating how to eliminate the poisonous substances from their useful roots. We treat them in detail, examining their current and past use, their geographical distribution in Italy, and their eventual toxicity. We obtained 343 matches, of which 198 were reliable (certain matches) and 145 possessed some degree of uncertainty (due to generic or collective vernacular names). Among the 198 certain identifications, 140 species are present in the AlimurgITA database (92 mentioned for Tuscany) and 58 are not; for bread-making there are only documentary traces of 53 species for Italy and 7 for Tuscany. Moreover, among the total 198 species, 84 showed some degree of hazard. Researching edible toxic spontaneous species allows: (1) investigation, from an unusual perspective, of a historical period in which the poor conditions of some social strata led to finding unusual solutions to food provision; (2) idea generation to re-enable potentially useful WEPs whose use has been lost. Making a virtue of necessity!

## 1. Introduction

The census of the alimurgical species of Italian flora has revealed a relevant contingent of taxa (1103) present in popular Italian cuisines [1]. These species, also called Wild Edible Plants (WEPs) or Wild Food Plants (WFPs), have diverse uses depending on local preferences and gastronomic traditions. A small number of WEPs/WFPs are used for baking, both as a seasoning and as a way to increase dough mass.

Current documentary sources link the use of these species—abundant, available and sometimes pleasant—to periods of extreme poverty that forced local populations to use these edible spontaneous species, making a “virtue of necessity” [2,3,4].

Historically, the first italian authors to deal specifically with this alimurgic topic and the reasons that led to the use of wild species (or non-canonical species of the cultivation systems of the time) for baking were two Tuscan authors: Saverio Manetti in his treatise of 1765 [5] and Giovanni Targioni Tozzetti in his text of 1766 [6]. Both of them wrote explicitly about the famine events that occurred in Tuscany in 1764 and 1765 and dedicated their efforts to two works dealing with unusual vegetable products used in bread making. There are, however, substantial differences in the approach they chose for treating this topic.

Manetti mainly focused his attention on the different species of cereals or exotic plants successfully used by local people, relegating the reference to spontaneous species to a small section of his extensive book. In contrast, Giovanni Targioni Tozzetti in his text published a year before the more famous treatise *Alimurgia* [7], in addition to different cereal species, shifted the focus to the use of specific wild plants to “increase the mass of bread”, that is, to increase the amount of flour for bread making with wild plants that today would be defined as “alimurgic”.

Giovanni Targioni Tozzetti (Florence, 1712—Florence, 1783) in the year 1734 graduated in “medicine and natural philosophy” with a thesis on the effectiveness of the use of plants in medicine (*De praestantia et usu plantarum in Medicina*) [8] but his research was applied, as often happened at the time, to many other areas of knowledge (botany, agronomy/agriculture, geology, mineralogy, paleobotany, human history, zoology, etc. [9,10,11,12]). In his little book on bread-making, he addressed a rich repertoire of species, especially wild ones, as traditionally used in the Tuscan countryside probably integrating their study with consolidated information from other regions or nations. In the introductory section, the author himself incisively describes the state in which some social strata were living and the consequences to which they were subjected by the scarcity of food. Targioni Tozzetti, in a very direct and explicit manner, writes: “…*stimolati dall’arrabbiata fame, si pascono di sostanze nuocive o per loro natura, o per non le aver sapute abbonire, e spogliare della qualità nemica; e così a poco a poco i miseri succhiano un Veleno a tempo, che col variare delle Stagioni, produce Malattie sterminatrici delle Popolazioni intiere*” (stimulated by angry hunger, they graze on substances that are harmful either by their nature, or for not having known how to abate them, and strip them of their enemy quality; and so little by little, the poor suck a poison in time, that with the variation of the seasons, produces diseases that exterminate the entire population) [6] (p. 3). Therefore, the risk of an improper use of toxic alimurgical species was well known, as well as the operations to be carried out to neutralize the poisonous substances in order to make such plants available for human consumption.

In order to deal with this particular aspect of alimurgic plants, we decided that the species for bread making identified in the work of Giovanni Targioni Tozzetti should be analyzed. His work is a valuable botanical document so far studied only from a historiographical point of view [13].

The specific aims of this work are many:

1. To bring to light, make available and analyze for the first time the only repertory of alimurgical flora compiled by Giovanni Targioni Tozzetti in Tuscan vernacular, matching those vernacular plant names with their current scientific names;
2. To compare the historical alimurgic repertory with the AlimurgITA database (data for the last 100 years) of Italy; making it possible to develop hypotheses on the causes of the survival or disappearance of the use of some WEPs;
3. To highlight the use of toxic edible plants as recognized by Giovanni Targioni Tozzetti [6]. The list has been critically screened and updated with recent toxicologic acquisitions, prompting systematic investigation, for the first time, of Italian toxic WEPs;
4. To describe, from a botanical perspective, a historical period in which the last famines in Italy occurred;
5. To provide ideas for the use of WEPs in the field of modern baking.

## 2. Materials and Methods

In the text of Giovanni Targioni Tozzetti: “*Breve istruzione circ’ai modi di accrescere il pane col mescuglio d’alcune sostanze vegetabili*” (Brief instruction on ways to increase bread using mixtures of vegetable substances) [6], for the species mentioned for use in baking, Tuscan vernacular names from the 18th century were used. In order to avoid, as much as possible, taxonomic mistakes, we first consulted the 1858 edition of the *Dizionario botanico italiano* [14,15] edited, starting from 1809, by Ottaviano Targioni Tozzetti, son of Giovanni. The first volume of the dictionary lists the names of the plants in Tuscan vernacular with the corresponding Latin name in Linnaean binomial; the second volume proceeds in reverse, listing the Linnean binomials and associating their vernacular names. Our approach seemed the best both for the temporal proximity with the writing of Giovanni Targioni Tozzetti and for an inherent coherence with the references to the same plants made by the two authors.

However, for some of the vernacular names cited by Giovanni Targioni Tozzetti, consultation with the *Dizionario botanico italiano* [14,15] showed a lack of correspondence or a doubtful attribution. The same vernacular name can correspond to more than one species even if not taxonomically close. Furthermore, there are “collective” names, including several species often declined in the plural form: *gramigne*, *canne*, *biodi*. In an attempt to fill these gaps, to confirm the possible connections between the vernacular and the scientific names, we also consulted the *Dizionario delle scienze naturali* published in 22 volumes from 1830 to 1851 [16]. The *Dizionario delle scienze naturali* counted Antonio Targioni Tozzetti (nephew of Giovanni) among its authors, as an Italian collaborator expert in botany.

In order to compare to current Italian ethnobotanical knowledge, each plant name in Giovanni Targioni Tozzetti’s list has been associated with the latest nomenclature system for Italy [17,18] and paired, whenever possible, with the corresponding records in the AlimurgITA database.

The toxic species mentioned are described with the following characteristics:

- their use according to Giovanni Targioni Tozzetti [6];
- their use according to Mattirolo’s *Phytoalimurgia Pedemontana* [19]. In this book, he provided a wide and reasoned repertory of WEPs used in Italy in the early 1900s, including toxic ones, with valuable information on their use and transformation. He is considered the father of modern Italian alimurgia as he was was responsible for redefining this branch of ethnobotany;
- definition of their toxicity according to the current toxicological literature;
- their possible presence in the AlimurgITA database.

For the other species in Giovanni Targioni Tozzetti’s repertory, not explicitly considered harmful, possible toxicity has been assessed using the most recent, national, ethnobotanical work for Italy [20], Acta Plantarum website [18], and the Plant for a Future—PFAF website that contains information on the use of plant species globally [21].

Finally, by comparing the list of species by Giovanni Targioni Tozzetti with those in the AlimurgITA database, we were able to identify the number of species in the repertory that are also in our database and if some of them were not mentioned.

## 3. Results

### 3.1. The Alimurgic Repertory of Giovanni Targioni Tozzetti

The work of Giovanni Targioni Tozzetti identifies “*sostanze vegetabili*” (vegetable substances) that can be added to the usual flours (wheat, rye, barley, etc.) to make bread or polenta, *necci* or *castagnacci*. These vegetable substances are divided into four categories [6] (p. 3): (1) “*Midolle o anime di semi*” (medullae or souls of seeds—paragraphs I to XXIII), (2) “*Polpe corticali, o Scorze sugose e morvide di frutti*” (cortical pulps, juicy and soft peels of fruits—paragraphs XXIV to XXX), (3) “*Radiche*” (roots—paragraphs XXXI to XXX), (4) “*Foglie, Cime, Cortecce, Rami teneri di Piante*” (leaves, tops, barks, tender branches/young shoots of plants) defined as “*l’infimo rango di Alimento Vegetabile*” (the lowest rank of vegetable food) (paragraphs XXXIX to XLIV) [6] (p. 12). A separate section is devoted to substances suitable for flavoring or modifying flavor (although these uses are also mentioned elsewhere in the text) (paragraph XLV).

The lexical richness of his work is remarkable. There are a total of 342 vernacular names of plants (often more than one is used to indicate the same entity or different species are indicated with the same name), with 21 that we considered “collective names”, i.e., those that are used, mostly in the plural, to indicate at least two or more species (*Abeti*, *Biodi/Biodri*, *Canne*, *Cardi o Scardiccioni*, *Carici*, *Cipperi*, *Cipperoidi*, *Giunchi*, *Gramigne*, *Ieracj*, *Latiro*, *Querce*, *Rose domestiche*, *Rose salvatiche*, *Salci*, *Scorzonere/Scorzonera*, *Sedi o Semprevivi*, *Trifoglio*, *Vecce*, *Vecce Salvatiche*, *Zucche*). It was a challenge to interpret and differentiate plural names used to indicate a group of species from names used to indicate a single species declined in the plural, e.g., *Cicerchie* (*Lathyrus sativus*) or *Veggioli* (*Ervilia sativa*).

The operation of matching vernacular and scientific names led to a list of 343 possible species (Supplementary Materials Table S1). However, it was not possible to attribute one or more species to some of the vernacular names (Supplementary Materials Table S2):

- “*Medicaggine*”: could go among the collective names, because Ottaviano Targioni Tozzetti identified several possible *Medicago* species [14,15]. In his previous work *Istituzioni botaniche del dottore* he illustrated the *Medicaggine* legume citing the species *Medicago orbiculata* [22] (p. 612, figure 454);
- “*Cipperoidi*”: according to the *Dizionario delle scienze naturali* [16] it is a synonym of *Cipperi*, but Giovanni Targioni Tozzetti [6] uses both names in the same sentence as two distinct entities.

The following ones remain at the level of a collective name with no certain correspondence:

- “*Giunchi*”, “*Rose domestiche*”, “*Salci*”: because they correspond to many species;
- “*Latiro*” and “*Trifoglio*”: because these two, though declined in the singular, correspond to more than one species or genera.

As for the uses in bread making: 52 vernacular names are mentioned for seeds (corresponding to 70 possible species), 25 for fruits (27 possible species), 51 for roots (78 possible species), 138 for leaves and other parts of the plant (180 possible species), and 35 as aromatizers or flavor modifiers (44 possible species). Furthermore, in the section of flavoring plants 15 plants are identified for use against scurvy (19 possible species). The sum of citations may be greater than the number of species because some are cited for two or more uses.

Among the 343 possible species, 298 are spontaneous and 45 cultivated. In 70 cases, the correspondence is not one to one, because more than one species can correspond to a collective or generic vernacular name, or more than one vernacular name can correspond to a species. For example, *Cardi o Scardiccioni* corresponds to 14 binomial names (*Carduus nutans*, *C pycnocephalus*, *Carthamus lanatus*, *Centaurea solstitialis*, *Cirsium arvense*, *C. oleraceum*, *Drypis spinosa*, *Echinops sphaerocephalus* subsp. *sphaerocephalus*, *E. strigosus*, *Genista germanica*, *Ptilostemon stellatus*, *Scolymus hispanicus*, *S. maculatus*, *Silybum marianum*), *Erba Kali* corresponds to 3 binomial names (*Salsola kali*, *S. tragus*, *Soda inermis*), *Cipperi* corresponds to 2 binomial names (*Cyperus longus*, *C. rotundus*), *Malva* corresponds to 2 binomial names (*Malva neglecta*, *M. sylvestris*), *Ortica* corresponds to two binomial names (*Urtica dioica*, *U. urens*), etc. This led to the listing of 145 “uncertain species”, which we do not discuss in detail except in one interesting case. It regards two possible species suggested to give a salty taste to dull doughs. It is the algae *Fuchi marini* which is probably *Fucus vesiculosus* and *Sargassum natans*. Both species today are reported to have an oceanic distribution, but in the *Dizionario delle scienze naturali*, they are reported as present also in the Mediterranean [16]. In particular, the *Dizionario delle scienze naturali* suggests that *Fucus vesiculosus* is the “quercus marina” of the ancients (e.g., Plinio) [16] (vol. XI, part II, p. 1204).

**Figure 1:**
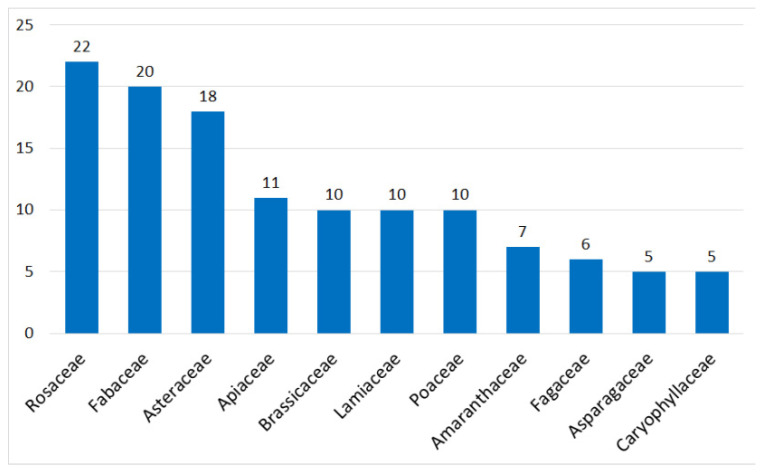
Families represented by more than five species for the 198 certain species.

**Figure 2:**
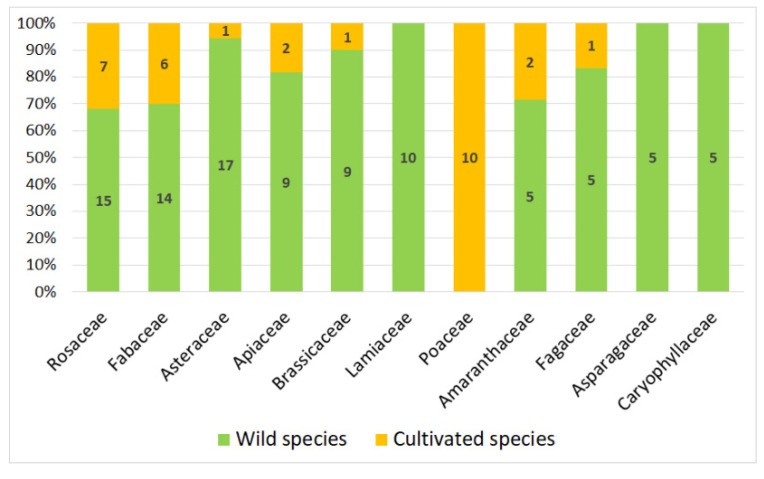
Most represented families: distribution of the 30 cultivated species.

There are 198 vernacular names with a clear corresponding single modern species (158 wild species and 40 cultivated), distributed in 59 families. Eleven families are represented by at least five species (Figure 1) and among them those with the highest number of species are Rosaceae (11.11%), Fabaceae (10.10%) and Asteraceae (9.09%). These eleven families contain 30 of the 40 cultivated species, of which one-third are Poaceae (Figure 2).

**Figure 3:**
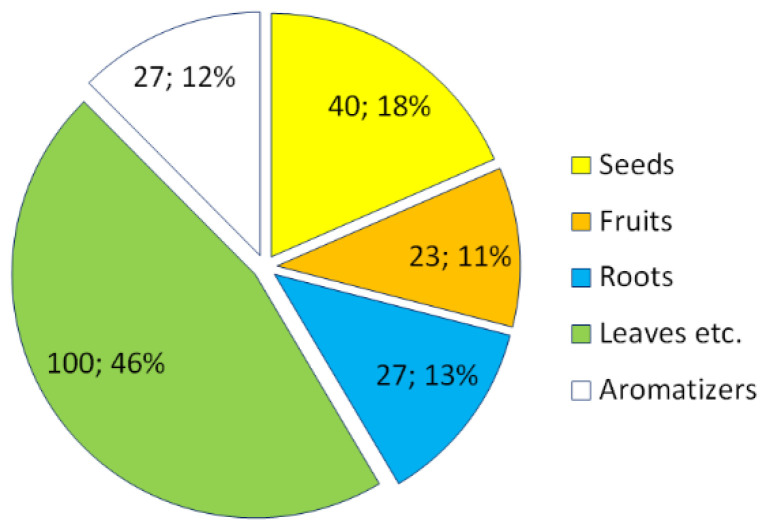
Frequency of citation of the 198 species in the sections of the work of Giovani Targioni Tozzetti [6].

Regarding their uses, the 198 certain species are mostly mentioned concerning the uses of leaves and other parts (raw if tender, cooked if harder or of unpleasant taste), and secondarily in the paragraphs dealing with the uses of seeds (Figure 3).

**Figure 4:**
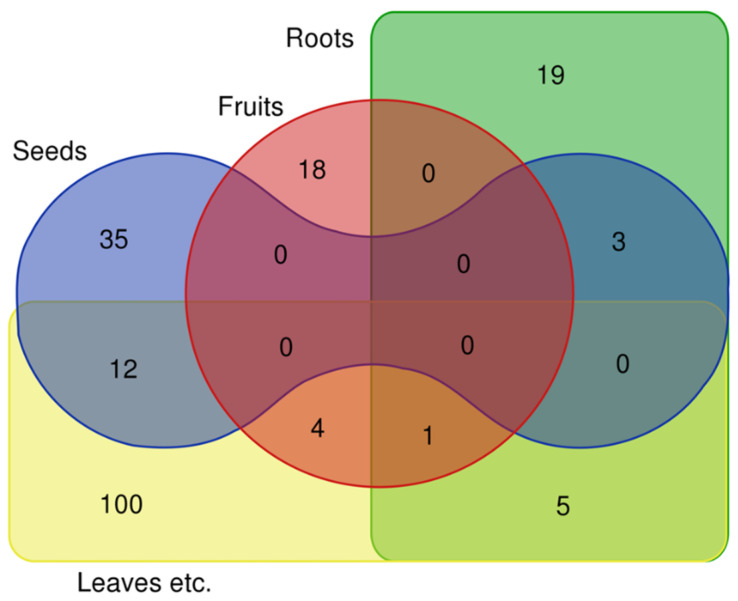
Venn diagram of the parts used of 197 certain species (the omitted species, *Sambucus nigra*, is the only one mentioned only for flowers and was not included in the diagram).

As for the parts used (Figure 4), these are mainly the leaves and other parts (122 species), followed by the seeds (50 species), roots (28 species), fruits (23 species), and flowers for only two species (*Sambucus nigra* and *Vitis labrusca*).

### 3.2. Comparison with the AlimurgITA Database

Among the 198 “certain species”, 58 are missing from the AlimurgITA database, but 23 of these are cultivated, thus excluded *a priori* from the database [1], and one species is a lichen (*Cetraria islandica*, Parmeliaceae). This leaves us with only 34 truly absent species: *Acanthus mollis*, *Althaea cannabina*, *Angelica archangelica*, *Anthyllis vulneraria*, *Asplenium sagittatum*, *A. scolopendrium*, *Caltha palustris*, *Clinopodium nepeta* subsp. *spruneri*, *Cruciata glabra*, *Drabella muralis*, *Dryopteris filix-mas*, *Fraxinus excelsior*, *Gagea lutea*, *Galatella tripolium*, *Jacobaea vulgaris*, *Limniris pseudacorus*, *Lysimachia nummularia*, *Medicago arborea*, *Melampyrum arvense*, *Narcissus pseudonarcissus*, *Paeonia officinalis*, *Phillyrea latifolia*, *Pimpinella anisum*, *Populus alba*, *P. nigra*, *P. tremula*, *Potentilla erecta*, *Pyracantha coccinea*, *Rorippa palustris*, *Senecio ovatus*, *Sium latifolium*, *Solidago virgaurea*, *Tamarix gallica*, and *Viburnum tinus*.

**Figure 5:**
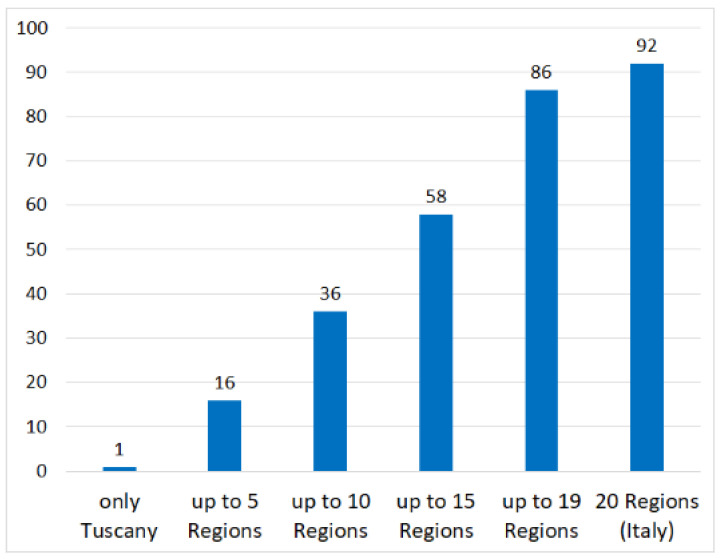
Distribution at Italian regional scale of the 92 species of the AlimurgITA database mentioned by Giovanni Targioni Tozzetti [6] and still used in Tuscany.

Among the 140 entities present in the AlimurgITA database, 92 are still used in Tuscany: one is used exclusively in Tuscany (*Eupatorium cannabinum*), 15 are used in up to 5 regions, 20 are used in up to 10 regions, 22 are used in up to 15 regions, 28 are used in up to 19 regions and 6 are used in all Italian regions (*Borago officinalis*, *Humulus lupulus*, *Nasturtium officinale*, *Rumex acetosa* subsp. *acetosa*, *Silene vulgaris*, *Taraxacum officinale*) (Figure 5).

The comparison with the AlimurgITA database extended to Italian WEPs used for bread making that are documented in the ethnobotanical literature of the last hundred years. These species were divided into two categories according to their use (plants for the production of flour and bread flavoring) and the parts used were considered. Overall, 53 taxa were identified, of which 35 were used for baking and 19 as flavoring (*Elymus repens* subsp. *repens* has a dual use) (Supplementary Materials Table S3). For eight species (*Bistorta officinalis*, *Brachypodium sylvaticum* subsp. *sylvaticum*, *Elymus repens* subsp. *repens*, *Ervilia sativa*, *Lathyrus sylvestris* subsp. *sylvestris*, *Lolium pratense*, *Nymphaea alba*, *Quercus pubescens* subsp. *pubescens*) their use was specifically linked to periods of famine and is therefore considered as survival use that is most likely no longer practiced today.

**Figure 6:**
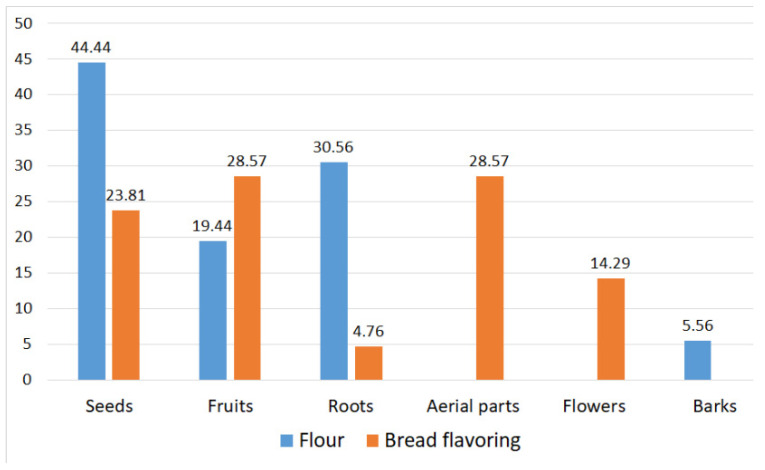
Parts used for the production of flour and bread flavoring of 53 alimurgic taxa present in the AlimurgITA database.

The same classification criterion (flour or bread flavorings) was used for the parts used for bread making to allow comparison between the two data sets (AlimurgITA database and Giovanni Targioni Tozzetti’s repertory). The results show that the seeds and the hypogeal part constitute the predominant percentage in flour production (44% and 31%, respectively) while among the flavorings the values of the fruits, seeds, and leaves are similar (28%, 24%, and 24%, respectively) (Figure 6).

The list of species from the AlimurgITA database were compared with the one proposed by Giovanni Targioni Tozzetti (198 species), finding only 37 taxa in common (Table S3, Section A). The remaining 16 (Table S3, Section B) are linked to very limited regional uses; for example, *Brachypodium sylvaticum* subsp. *sylvaticum* for Emilia–Romagna [23], *Betula pendula*, *B. pubescens*, *Erythronium dens-canis* for Veneto [2], and *Nigella arvensis*, *Schoenoplectus lacustris* for Apulia [24].

### 3.3. The Toxic Plants

Among the spontaneous plants, eight were identified as toxic by Giovanni Targioni Tozzetti [6] (p. 11). They provided the starting point for verifying their regional and temporal use to date, by consulting the database AlimurgITA. For each of the corresponding 14 species we have provided a brief ecological and chorological description, an indication of their use, and a comparison with toxicity information from the most recent literature to confirm or not the toxicity information provided by Giovanni Targioni Tozzetti [6].

Details of the procedures for the removal of the toxic substances, according to Giovanni Targioni Tozzetti, from the roots of ungrateful or harmful flavors (Preparations of roots “*di sapori assai acuti, ingrati, ed anche nuocivi*”—of very sharp, unpleasant, and even harmful flavors) [6] (p. 11) are reported in Table 1.

#### 3.3.1. “*Aro*, *Gichero*”—*Arum italicum*, *A. maculatum* (Araceae)

*Arum italicum* and *A. maculatum* (also called in Italy *Gigaro* or *Pan di Serpe*) are perennial forbs native to the European subcontinent. Four subspecies are recognized and their combined native range extends from Portugal in the west to Georgia in the east, south to Morocco and Algeria in North Africa, and north to the southern edge of England [25].

Both species of *Gigaro* are distributed in every Italian region except for *Arum maculatum* which is absent in Sicily. Ecologically similar, the two species grow in forest environments but also along ditches, roads, hedges and cultivated areas (vineyards and olive groves) from 0 to 800 (1000) m above sea level.

The Gigaro has been used as food in different parts of Europe especially for the starch extracted from its tubers, for the preparation of bread. In England, the starch extracted from *Arum maculatum* was very popular and known as “Portland sago”, and in the Czech Republic the rhizome was used particularly in times of famine ground into flour [26]. In Bosnia the tubers of *Arum italicum* and *A. maculatum* are still used for the preparation of boiled meats or focaccia [27]. In Albania, the use of *Arum italicum*, which was eaten during periods of famine, is documented and its use has been preserved to this day, albeit in different ways [28]. The leaves are also consumed in southeastern Europe after repeated boiling. In Switzerland, *Arum* leaves are eaten in spring as a purifying cure [29].

In Italy *Arum italicum* and *A. maculatum* are mainly considered for their officinal use, finding application in several diseases such as hemorrhoids [30,31], furuncles [4], chilblains [32], leeks [33,34], arthritis [35], contusions [36], rheumatism [37,38,39], varicose ulcers [20]. Leaves of *Arum italicum* in poultice are applied for wound healing in Tuscany, Campania, Basilicata, Sicily, and Sardinia [40].

Locally (Tuscany), *Gigaro* is used as an analgesic or purgative [41]. Leaves and rhizomes of *Arum italicum* are also useful in making animal feed [33,38,42,43,44] or for domestic use.

Popular use for human nutrition was identified for Veneto [2], Liguria [45], Tuscany [46,47], Puglia [24] and Basilicata [1], although its current use cannot be verified. The part used is the ovoid, tuberiform rhizome, which has found its exclusive use in the extraction of starch for the preparation of baked goods (bread and focaccia). All the regional reports refer to its use contingent to the period of scarcity of food availability and it does not seem to find a current use.

The use of *Gigaro* was documented in human nutrition by Castore Durante in 1585; Durante reported that, in addition to its therapeutic qualities (“*scaccia i parassiti, chiude le ferite, favorisce le mestruazioni ma brucia*”—it drives away parasites, closes wounds, helps menstruation but burns”), it can be obtained from the rhizome of the flour which he praised as “marvelous” [48].

Giovanni Targioni Tozzetti warned his readers that the plant should be used only in extreme cases because of its “burning” taste [6] (p. 11), he nevertheless believed that it could be made harmless and available through a specific procedure of detoxification (Procedure 2, Table 1).

Mattirolo [19] did not consider *Arum italicum* and *A. maculatum* to be toxic, even if he strongly recommended boiling or roasting the rhizome in order to eliminate the caustic substances. He considered them very interesting plants because it is possible to obtain a very high quality starch comparable to rice, provided a specific protocol is strictly followed [49]. At the same time he reported the use of Gigaro starch in Sicily to make sweets and confetti or to flour figs, however, the use of Piedmont was not mentioned.

Tubers contain high amounts of sugar and starch (especially, two-year-old *A. italicum* tubers which contain up to 20% starch) [50]. Despite being recommended for use as food and medicine, the toxicity of *Arum* is indicated in most texts [51]. It is poisonous when fresh, effects of its toxic substances are lost when dried and boiled up.

The toxicity of *Arum italicum* and *A. maculatum* stems from the presence of Aroin, saponins, and cyanogenic compounds [52]. In addition to these, calcium oxalate, one of the main toxic compounds in *Arum* plants [53], and cyanoglycosides such as trigloquinine [54] are decomposed by cooking. Lastly, fractions of *Arum italicum* tuber extract were evaluated for their cytotoxic, antiproliferative and apoptotic effect by in vitro models [55].

The use of fresh plant, if ingested, causes serious gastro-intestinal disorders with nausea, vomiting, diarrhea, and heart rhythm disorders, and it may cause poisoning that leads to death. In applications for external use it causes skin inflammation. The toxic phytocomplex is present in every organ of the plant, but it has its highest concentration in berries [55].

#### 3.3.2. “*Asfodelo*, *Astula Regia*, *Porreca*, *Porraccio*”—*Asphodelus ramosus* L., *Asphodelus microcarpus* Salzm. et Viv., *Asphodelus albus* Mill., *Asphodeline lutea* (L.) Rchb. (Asphodelaceae)

These three species of asphodel take on different names in the Tuscan dialect. The *Porraccio* or *Porracci* (as called in the Tuscan vernacular, in relation to the tuberous roots), correspond to two species considered edible from the genus *Asphodelus*: *A. ramosus* and *A. albus* [14,15,56]. Under the name of *Porreca* or *Porreche*, it is referred to *Asphodelus fistulosus*, while with the name of *Asfodelo* or *Astula* regia, they are called both *Asphodelus ramosus* and *Asphodeline lutea* [14,15]. The latter is addressed in the next subsection.

*Asphodelus ramosus* is a species with steno-mediterranean distribution and is present in all the regions of central-southern Italy (except Marche), in Emilia-Romagna, and Liguria; *Asphodelus albus* has a Mediterranean-Montane chorotype and its distribution is fragmentary, being found with certainty in Piedmont, Lombardy, Friuli Venezia-Giulia and Apulia; and *Asphodelus fistulosus* is found in the whole Italian territory with the exception of Aosta Valley, Piedmont and Veneto. Ecologically, the three species prefer stony soils in uncultivated areas, in garrigues, and arid pastures, indicating with their presence areas of environmental degradation often linked to overgrazing.

*Asphodelus albus* and *A. ramosus* were consumed at the time of the Greeks and Romans, who cooked the tubers under ash. In Europe, its use has been documented for France and several countries in southeastern Europe (Bosnia, Greece), especially in the past, where they have been consumed after boiling [29].

Many of the applications are in the field of Italian popular medicine; the three species of *Asphodelus* are recognized as having a curative effect on different disorders such as cough [4], sore throat [57], urinary tract infection [58], hemorrhoids [59], wounds [60,61], sores [60], eczema [58,59,62], skin inflammation [38,40,58], rheumatism [58], corns and chilblains [4,57,58], hair loss and sunburn [59]. In Tuscany, the rhizome of *Asphodelus albus* is used cosmetically to lighten skin blemishes [3].

Based on ethnobotanical documentation, dietary use of *Asphodelus ramosus* is known of in Lombardy [63], Tuscany [3,46,56], Campania [64], Apulia [65,66], Basilicata [67], Calabria [68,69,70], Sicily [71,72,73,74,75,76,77,78,79,80,81], and Sardinia [4].

Traces of *Asphodelus albus*, as an edible plant, have been found in Piedmont [82], Lombardy [20], Tuscany [83], Abruzzo [84], Campania [64], Calabria [69,70], Sicily [85] and Sardinia [4], although, the presence of this species is not marked in the regions of central Italy [86]. For *Asphodelus fistulosus*, its consumption is documented for Apulia alone [24].

Although the use of the rhizome of *Asphodelus albus* for baking is reported in a general manner [87]—as well as reported by Giovanni Targioni Tozzetti [6]—the comparison with the Italian alimurgical literature has not confirmed its use in this way, which, therefore must be considered as abandoned. A similar use of *Asphodelus albus* for food was also found in French history, where the hulled rhizomes were consumed cooked or dried to obtain flour for bread making in times of famine [88,89].

Also, the inflorescences—the very tender branched panicles that have not yet flowered—are consumed, after blanching, with pasta or as an ingredient for omelettes (Piedmont, Calabria) [70]. A singular use of *Asphodelus albus* was reported by Mattirolo [19] for Piedmont and by Riccardo for Apulia [89], where the wide distribution of this species seems to have encouraged its wide industrial use for the preparation of alcohol.

The plant is toxic due to the presence of several alkaloids [90]. The roots mainly, were reported to have anthraquinone derivatives (chrysophanol and aloe-emodin, triterpenoids, and naphthalene derivatives), while the aerial parts contained mainly flavonoids such as luteolin, isovitexin and isoorientin, phenolic acids, and a few anthraquinones [91].

Fatty acids, namely myristic, palmitic, oleic, linoleic, and linolenic, were found in the seeds and roots. Some recent ethnopharmacological studies [92] have also revealed antioxidant and enzyme inhibitory activity from *Asphodelus albus* root extracts which attest to the presence of aloin A, aloin B, and aloesin suggesting the potential for new pharmacophores to alleviate oxidative stress-related complications, obesity, and skin hyperpigmentation complications [92].

*Asphodeline lutea*, called yellow-flowered asphodel, in the Tuscan dialect, or *Astula regia*, is a perennial plant typical of dry grasslands on poor and stony soils up to 1700 m and is widespread in most Italian regions with the exception of Valle d’Aosta, Piedmont, Veneto, and Trentino Alto-Adige.

In popular uses, the asphodel with yellow flowers is considered exclusively as a food plant that is consumed in Campania [93,94], Puglia [24,65,95,96,97], Calabria [70] and Sicily [72,79,80,98]. The not yet bloomed scape is consumed, still wrapped by the membranous sheaths of the leaves (called “*jannuli*” in Calabria and “*ggiummu*” in Sicily), and also the young leaves. In Apulia the stalks of the yellow asphodel (called “*avuzze*”, “*aveluzze*”, “*averusce*”), harvested before the flower blooms, are blanched with water and vinegar and preserved in oil. Leaves are still used to make burrata, a typical cheese product of Puglia. In some areas of Sicily, the stalks of yellow asphodel are harvested, deprived of external leaves, cut in half, and cooked with a sauce or in omelettes [87].

Another edible part of *Asphodeline lutea* is the root, which is usually boiled and seasoned with salt, oil, and vinegar, or mixed in soups.

Targioni Tozzetti recognizes the rhizome as the useful part for baking from which toxic substances must be eliminated through Procedure 1 (Table 1). Currently there is no trace of this use in the Italian alimurgic tradition, not even in Tuscany, the region from which the report comes.

Leaves, stems, and tubers contain flavonoids (apigenin, luteolin, kaempferol), benzene/naphthalene and anthraquinone derivatives (chrysophanol, aloe-emodin, isochrysophanol, physcion, rhein), compounds which can be responsible for gastroenteritis and whose intensity and gravity depends on the quantity consumed [99]. The plant should not be consumed raw and only immature stems should be harvested and eaten.

#### 3.3.3. “*Felce femmina*”—*Athyrium filix-femina* (L.) Roth (Woodsiaceae)

The female fern is a perennial herbaceous plant with a thick rhizome with sub-cosmopolitan distribution. Present in every Italian region, it usually lives in forest environments, in damp ravines, and sinkholes, sometimes even in humus-rich damp pastures from 0 to 1800 m above sea level.

*Athyrium filix-femina* is a species with low frequency in popular uses; its officinal uses are found only in Trentino Alto-Adige where the rhizomes, collected in winter and prepared in decoctions, were used to treat rheumatism [100,101] and neuralgia [101].

As an edible species, its use is limited to Apulia [24] and Basilicata [102] where the rhizome or young shoots are used and consumed boiled in soups. The young sprouts are considered in Sardinia as food flavoring substances [4].

Giovanni Targioni Tozzetti [6] informs the reader that the rhizome of this fern should be used only in cases of extreme need due to its unpleasant taste and toxicity, but can be rendered harmless by following Procedure 1 (Table 1).

In the few scientific contributions published so far, *Athyrium filix-femina* exhibited moderate to high cytotoxicity [103]. The results indicate that this plant could be toxic for humans, so caution must be taken when using this fern. In general, the toxicity of the leaf extracts is significantly higher than those of the rhizome extracts [103,104].

#### 3.3.4. “*Ciclamino*, *Panterreno*, *Panporcino*”—*Cyclamen repandum* Sm., *C. hederifolium* Aiton (Primulaceae)

*Cyclamen repandum* and *C. hederifolium* (called spring cyclamen and ivy-leaf cyclamen in Italian, respectively) are two North Mediterranean gravitational species present in all regions of central-southern Italy. They grow preferentially in *Quercus ilex* and deciduous woods (oak, chestnut) in deep soil in shady areas. The two species of cyclamen have in common the fact that they are sought after as food by pigs and ungulates, hence, the vernacular Tuscan name of *Panterreno* or *Panporcino*.

In the officinal field, cyclamen are widely used in Italy for the resolution of different pathologies. It is considered an emmenagogue remedy [105], digestive [37], anti warts [106], and anti chilblains [107]. In the form of oleolite, it is used for the treatment of otalgia [41,105,108], hemorrhoids [38,41], and arthritic pain [41]. In veterinary phytoiatry, *Cyclamen repandum* is considered an abortive plant [109].

In the text by Giovanni Targioni Tozzetti [6], the *Panterreno/Panporcino* is recognized as being used for baking; since it is a generic name, it is not possible to determine with certainty whether both species of cyclamen or one in particular was used. At the time, only the species *Cyclamen europaeum* was recognized. The author however recognizes their toxicity and indicates Procedure 1 (Table 1) as a way of neutralizing these substances and at the same time making the consumption of flour more pleasing to the palate.

In the work *Phytoalimurgia pedemontana* [19], Mattirolo reported the alimentary use of *Cyclamen repandum* and *C. hederifolium* because they are considered harmless after roasting, as suggested in the French experience by Cornevin [110]. In Italian food literature from the last one hundred years, cyclamen is rarely used as a food only in Lombardy [1] and Calabria; in the latter region it has been preserved in a restricted territory (Magisano, CZ) as a feculiferous plant [111].

Several species belonging to the genus *Cyclamen* L. (Primulaceae) are widely used in traditional medicine for their biological properties, but few investigations have been performed on this plant.

All cyclamen plants are dangerous because the whole plant and especially the fresh tuber contain the glucoside cyclamen, a highly toxic compound [112]. Phytochemical screening of *Cyclamen* species has revealed that tubers of this plant are rich in triterpenoid saponins [113]. The cytotoxic, spermicidal, antimicrobial, analgesic, and anti-inflammatory properties of *Cyclamen* saponin have been demonstrated in pharmacological studies [113,114,115]. These properties have also recently been recognized as having antibacterial [116] and anti-inflammatory activity [117].

#### 3.3.5. “*Felce maschia*”—*Dryopteris filix-mas* (L.) Schott (Dryopteridaceae)

The male fern is a perennial pteridophyte with a short and robust rhizome, surrounded by brown lignified scales. It is a sub-cosmopolitan species frequent in the woods of broad-leaved trees of the hilly and mountainous plains of all Italian regions.

In popular tradition, *Dryopteris filix-mas* finds diverse usage in human medicine, although it is now largely abandoned. There are few documents about its food use. During the 17th century famines in Europe, the *Dryopteris filix-mas* rhizome was probably used to make bread. It has recently been used in Bosnia and Siberia, to give beer a raspberry flavor [29].

In Italian ethnobotanical documentation, rhizomes and leaves are used as decoctions in the treatment of rheumatism [101], colitis [36], menstrual disorders [39], sciatica [100], neuralgia [101], arthrosis [107], hemorrhoids [36], goiter [118], and gout [119]. It is also considered a diuretic species [120] and anthelmintic due to the ability of filicin to paralyze tapeworm muscles [19,36].

Despite its wide use in the officinal field, no food use is currently reported for the male fern.

As for the female fern, Giovanni Targioni Tozzetti [6] recommended the use of the rhizome of this species only when absolutely necessary. In fact, it was considered to have an unpleasant and noxious taste, so the feculiferous part had to be obtained profitably through Procedure 1 (Table 1).

Mattirolo [19], following the indications of Duchesne [121], recommended the cooking of its young leaves, used as asparagus and reported the use of the rhizome in Auvergne (France) for bread-making, although he does not hide his skepticism due to the bitter and astringent taste of this plant.

Few data concerning the chemical composition of *Dryopteris filix-mas* are available. However, it seems appropriate to extend a certain caution, this species, because a certain number of ferns (e.g., *Pteridium aquilinum*) contain carcinogenic substances [122]. The fresh plant contains thiaminase, an enzyme that deprives the human body of its vitamin B complex. If taken in small amounts, this enzyme will not harm people who eat a vitamin B-rich diet; large amounts, however, can cause serious health problems. The enzyme is destroyed by complete drying or by high temperature, therefore cooking the plant has the ability to remove thiaminase [123].

Also, in this fern triterpenoid hydrocarbons are present in the aerial parts, together with several sterols. The main component is sitosterol [124] and plant extracts containing sitosterol are known to exhibit anti-inflammatory properties [125].

#### 3.3.6. “*Iride montana*, *Giaggiuolo*”—*Iris florentina* L., *I. germanica* L. (Iridaceae)

In the collective name *Iride montana* or *Giaggiuolo*, Giovanni Targioni Tozzetti [6] could be referring to two related species that have a dissimilar ethnobotanical use.

They are herbaceous perennial plants with fascicled roots and fleshy creeping rhizomes with a scent. Considered as adventitious species, in Italy they have found a different degree of diffusion. *Iris germanica* occurs in every Italian region, while *Iris florentina* is geographically limited to Lombardy, Tuscany, Marche, Latium, Campania, Calabria, Sicily, and Sardinia. Considered an ornamental species, probably as a consequence of its spontaneity, it is found not only in gardens but also in prairies of the hilly plains, on warmer slopes, and even stony ones.

*Iris florentina* and *I. germanica* have been used since ancient times to flavor culinary and alcoholic preparations (e.g., gin) or simply as a pleasant masticatory. When a wine has a musty smell in the barrel, an iris rhizome can be immersed in it to eliminate the smell. Traditionally in Chianti (Italy) the wine was flavored with *Iris florentina* rhizome powder to give it a scent of violets [29].

*Iris florentina* (called *Giaggiolo biancastro*, in Italian) is used as an officinal plant in the treatment of myalgia [126], rheumatism [126], or for eye diseases in the form of eye drops [3]. No use in food preparations is reported.

Plants can cause skin irritations and allergies in some people [127]. The leaves, and especially the rhizomes, of this species contain an irritating resinous substance called irisin. If ingested, irisin can cause severe gastric disturbances [21,128].

*Iris germanica* L. or sword-lily iris (called *Giaggiolo paonazzo* or *giaggiolo germanico*, in Italian), was a plant held in high esteem for its versatility due to its contemporary medicinal, cosmetic, alimentary, and ornamental qualities.

In the preparation of traditional medicines in Italy mainly the rhizomes of this plant are used to relieve gastric pain [129], cough [38,129], or as emetic [38], or diuretic [119]. As an ointment it is used in local antirheumatic and antimialgic rubs [3,126], and against headache [129]. To mitigate teething pain in children it was recommended to chew dry and clean pieces of rhizome [119,129,130]. In Tuscany only, it was used as eye drops for ocular diseases [3].

*Iris germanica* is considered an edible plant in Lombardy [63], Tuscany [3,131], and Apulia [24]. The part considered edible is the rhizome, cleaned and cut into slices, that are consumed fried; although consumption at high doses can cause nausea and vomiting.

Mattirolo [19] did not consider it a useful plant although he points out that from the rhizomes a good quality starch can be achieved, whose extraction is not economically convenient because of the need to use long and specific procedures/treatments.

A few years later, Riccardo [89] reported that from the rhizome a good quality starch can be obtained, and from the roasted seeds, a coffee substitute can be produced.

The sword-lily iris has been cultivated since ancient times, and its rhizomes have been collected because they were known to contain, increasingly on storage, aromatic substances that were used to prepare perfumes and cosmetics with the fragrance of sweet violet, an essential oil that emanates a strong smell of violet (irone), soft resin (camphor of Ireos), yellow coloring matter, aldehydes, esters of various acids, a ketone, and a glucoside (iridine) [132].

The plant rhizomes contain several types of quinones and quinols, flavonoids, isoflavonoids and their glycosides (iridin), benzoquinones, triterpenoids, and stilbene isoflavone glycosides [133,134].

In the light of recent scientific discoveries, it does not appear that *Iris germanica* has toxic substances in the rhizome, thus disputing its evaluation as a harmful species carried out by Giovanni Targioni Tozzetti [6].

#### 3.3.7. “*Iride palustre dal fior giallo*, *Pseudacoro*”—*Limniris pseudacorus* (L.) Fuss (Iridaceae)

*Limniris pseudoacorus* or Yellow-flowered Mountain Iris (Giaggiolo acquatico, in Italian) is a perennial herbaceous plant with an Eurasian chorotype and a large branched rhizome. This species grows in damp marshy areas, swampy woods, and in shallow water or wet ground on the edges of rivers and ditches. It is often found in shady places.

It is recognized as a medicinal plant in Tuscany for the treatment of myalgia and rheumatism [126], and for sternutatory use in Sardinia [39]. It therefore has a very narrow field of use. The fresh root is astringent, cathartic, emetic, emmenagogic, and odontalgic [135,136,137].

A slice of the root held against an aching tooth is said to bring immediate relief [138]. It was at one time widely used as a powerful cathartic but is seldom used nowadays because of its extremely acrid nature [135]. It can also cause violent vomiting and diarrhea [138]. When dried the root loses its pungent taste and then only acts as an astringent [135].

For human consumption, the cleaned and sliced rhizomes are used and generally consumed fried in both Tuscany [3] and Sardinia [4]. Ingestion at high doses can cause nausea and vomiting.

In addition to its harmfulness, *Limniris pseudacorus* was judged by Targioni Tozzetti as a plant with an unpleasant and acrid taste, whose rhizomes are usable in extreme cases of starvation provided that Procedure 2 (Table 1) is followed.

For human nutrition, Mattirolo [19] did not consider the use of rhizomes. He only mentioned the seeds which, if roasted, present a valid coffee substitute as was used in France and England [139].

Most of the compounds are mainly accumulated in the rhizomes such as quinones, cardiac glycosides (digoxin, digitoxin, ouabain) and isoflavones; the presence of phenolic compounds was mentioned for flowers and leaves [140,141]. The leaves, and especially the rhizomes, of this species contain an irritating resinous substance called irisin. If ingested this plant can cause severe gastric disturbances [128], skin irritations, and allergies in some people [127].

#### 3.3.8. “*Peonia*”—*Paeonia officinalis* L. (Paeoniaceae)

This is the case of a plant of doubtful attribution since it is mentioned by Giovanni Targioni Tozzetti [6] as *Peonia* and attributed by both Ottaviano Targioni Tozzetti [14,15] and *Dizionario delle scienze naturali* [16] to the species *Paeonia officinalis* which, is not considered edible in Italy. The generic name of *Peonia* could therefore be extended to two other species of Italian flora such as *Paeonia mascula* and *P. peregrina* present in the ethnobotanical literature and also limited to the officinal field. In fact, *Paeonia officinalis* is considered useful as cardiotonic [142], while *Paeonia mascula* and *P. peregrina* add to this property that of antispasmodic [142], sedative, and febrifuge [4,142].

These *Paeonia* species are perennials, herbaceous in appearance, with a woody rhizome at the base with roots formed by various spindle-shaped brown tubers. The ecology is similar because the three species of peony are found spontaneously in sparse woods, in subalpine shrubs, or along stony, dry, preferably calcareous, slopes. *Paeonia officinalis* is found throughout central-northern Italy while *Paeonia mascula* and *P. peregrina* seem to be vicariates of *P. officinalis*, usually distributed in the regions of southern Italy, with the exception of Molise.

Despite this limited use in Italy, the genus *Paeonia* is considered one of the most important crude drugs in Anatolic and Chinese traditional medicine. It was used against atopic eczema as well as for anticoagulant, anti-inflammatory, analgesic, and sedative purposes [143,144]. Some of the peony species in Anatolia have been consumed as tea for the treatment of constipation and epilepsy as well as for antitussive purposes [145].

Recent findings reveal an antioxidant property of substances and essential oils contained in the rhizomes of twelve *Paeonia* species, including *Paeonia officinalis* and *P. mascula* [146]. The three species have in common the presence of toxic substances which are localized especially in the rhizome that lead to intestinal and gastroenteric disorders or vomiting [147,148].

#### 3.3.9. Comparison between Recent Modern Toxicological Data and Giovanni Targioni Tozzetti’s Repertory

**Figure 7:**
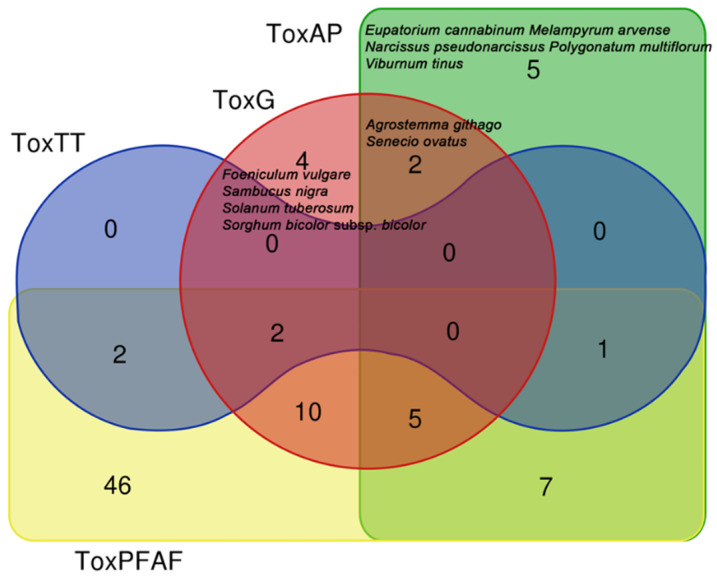
Venn diagram of the toxicity of 198 certain species considered toxic/harmful by Giovanni Targioni Tozzetti (ToxTT) [6], Acta Plantarum (ToxAP) [18], Guarrera (ToxG) [20], and Plants For A Future—PFAF [21] (ToxPFAF).

Considering the 198 species with a reliable attribution, but without deepening our knowledge on the level of toxicity (which depends on several toxical variables such as environment, part of the plant used and its maturation level, quantity of the toxic substance assumed, etc.), the species that are considered harmful for Plants for a Future—PFAF [16] (in relation to the part used by Giovanni Targioni Tozzetti [6]) are 73. If we add also the species considered toxic by Guarrera [20] and Acta Plantarum [18] (which do not report the toxic part) the number rises to 84 species. The comparison among the three sources of the data is reported in Figure 7.

Among the 84 species, we found:

- the five species mentioned by Giovanni Targioni Tozzetti [6] that have already been discussed (*Asphodelus albus*, *Athyrium filix-femina*, *Dryopteris filix-mas*, *Limniris pseudacorus*, *Paeonia officinalis*);
- five species considered toxic/hazardous by PFAF [21], Acta Plantarum [18], and Guarrera [20] simultaneously, twelve species considered toxic/hazardous by Guarrera [20] and PFAF [21], eight considered toxic/hazardous by Acta Plantarum [18] and PFAF [21] (Table 2);
- alimurgic species very often used in Italy (*Borago officinalis*, *Humulus lupulus*, *Nasturtium officinale*, *Rumex acetosa* subsp. *acetosa*, *Silene vulgaris*, *Chenopodium album*, *Clematis vitalba*, *Melissa officinalis*, *Ficaria verna*, and *Stellaria media* subsp. *media* limited to those cited for at least 16 Italian regions).

## 4. Discussion

In his booklet, the only text in which he speaks in such a precise and extensive way about alimurgical species, Giovanni Targioni Tozzetti manages to express the remarkable floristic diversity present in Tuscany with a lively and rich lexicon. His deep knowledge of floristry is not only about the plants that are consolidated in popular use, but also about those that have a potential use in the food field. We can perhaps say, without fear of being contradicted, that the small treatise on spontaneous species for bread-making is an ethnobotanical study *ante litteram* that derives not only from his interests and contacts with the peasant world and its tradition, but also from a thorough knowledge of the territory of the Grand Duchy of Tuscany (corresponding to the current regional limits of Tuscany). Proof of this can be found in the panorama of his works: “*Viaggi fatti in diverse parti della Toscana per osservare le produzioni naturali e gli antichi monumenti di essa*” (Travels made in different parts of Tuscany to observe the natural productions and the ancient monuments of it) [149], in which a description of Tuscany is made from both the historical and scientific point of view, ranging from geography and botany to medicine, zoology, mineralogy and architecture. Considering these premises, it is a pity that the second volume of the much better known treatise *Alimurgia* [7] was not published, which could have provided a great deal of insight on this subject.

The editorial style of the booklet on bread [6] is popular and practical because it is addressed to the poorest members of society who suffered the most from the terrible effects of famine. For this reason, Giovanni Targioni Tozzetti published at his own expense this short treatise that he reprinted shortly after, following its success.

The author, by concluding his writing, addresses all those who could read (parish priests, pharmacists, apothecaries, doctors, landowners, etc.) to do their utmost to spread these precepts to the poor people. Considering the target readership, it is interesting that the author had chosen to report in a lexically rich vernacular (342 different plant names) the species with only one name or with synonyms and collective names which, however, have created difficulties of taxonomic attribution and have sometimes remained unsolved. At the same time, the need to address a wide audience probably makes Targioni Tozzetti omit necessary references to other authors on similar uses of the species mentioned for other regions of Italy or nations as Mattirolo [19] and Riccardo [89] will do later in their works. Therefore, in our study it has not been possible to determine with certainty the number of alimurgical species of popular use in force in the middle of the 18th century, thus depriving us of information that would have been precious in order to understand the permanence or the disappearance of the use of some species in the Tuscan tradition.

Structuring his work, Giovanni Targioni Tozzetti organizes the species in categories according to their alimentary qualities; in this scale of values, the preferences are addressed to those parts of the plant (seeds, fruits, and amyliferous roots) that provide “chilo” such as to ensure, at the same time, quantity and quality of nutritive compounds. Despite being the largest number, the species of which only leaves, young shoots and stems could be used are of little interest to him and are therefore relegated to the lowest rank of vegetable foods (among those most used today are *Borago officinalis*, *Bunias ericago*, *Campanula medium*, *Cirsium vulgare*, *Lunaria annua*, *Portulaca oleracea*, *Prunella vulgaris*, *Reichardia picroides*, and *Taraxacum officinale*).

We paid particular attention to paragraph XXXVII in which eight toxic plants are described as “harmful” or toxic for human health and these represent the focal element of our work. These species were considered by Giovanni Targioni Tozzetti [6] as suitable for food only after some procedures that were used not only to detoxify them, but also to make their taste more palatable. In these procedures boiling water, ashes and/or cream of tartar (called “gruma di botte”) were commonly used as an empirical method to make thermolabile compounds harmless and to sequester/solubilize/precipitate in water some categories of toxic compounds, mainly alkaloids. According to the recent toxicological literature, of the eight toxic plants (corresponding to 14 species) mentioned in the Targionian repertory, some (e.g., *Iris germanica*) are not considered toxic while, on the contrary, 84 species with a reliable attribution considered as harmless, show a degree of toxicity. For example, Boraginaceae such as *Borago officinalis*, *Symphytum officinale* and *S. tuberosum* subsp. *angustifolium* contain unsaturated pyrrolizidine alkaloids [150,151], if used frequently, these plants have a hepatotoxic and blandly mutagenic action [87]; moreover, experiments on animals have shown their carcinogenic action, through a genotoxic mechanism [152]. Species with medium or low toxicity include those which appear dangerous only in cases of excessive and prolonged consumption, for example, those in the genus *Rumex* and *Oxalis* due to the presence of oxalates, or of *Amaranthus*, which can accumulate nitrates. It should be finally emphasized that, although it is an important element, if not fundamental, the quantities of these toxic foods that could be consumed without damaging the health are never reported, ignoring the famous motto of Paracelsus, “it is the dose that makes the poison”.

Are the two empirical methods designated by Giovanni Targioni Tozzetti [6] (Table 1) effective for detoxifying toxic plants?

The first procedure seems suitable for those species in which they are present:

- saponins (triterpenoid or steroid); the basic environment given by the ash (or the acid environment given by the gruma di botte—bees wing or cream of tartar) hydrolyzes the saponins, eliminating the monosaccharides and detoxifying the plant matrix;
- alkaloids; the ash makes the alkaloid no longer soluble in water, causing it to precipitate as a residue that can be eliminated;
- cyanogenic glycosides (e.g., amygdalin); the water, tartaric acid and boiling hydrolyze the nitrile to carboxylic acid. Generally, hydrolysis is not total but the concentration of these substances decreases by 60–80%. It is no coincidence that Giovanni Targioni Tozzetti [6] urges people to repeat the method.

The second procedure adds to the first: the shredding, drying, and grinding of the vegetable parts. The subsequent boiling in water of these parts therefore seems more specific for cyanoglycosides (present, for example, in *Arum italicum* and *A. maculatum*), which are soluble in water, to be hydrolyzed.

Obviously, neither the first nor the second method is infallible, mainly because it is not indicated what dosage of the substances should be used to modify the pH, nor the time of the procedure!

Another important result, previously mentioned, concerns the still intact cultural heritage of the food knowledge of the peasant world that is evident in the writing of Giovanni Targioni Tozzetti [6]. This led us to compare these data with those of the AlimurgITA database in order to hypothesize how many species have disappeared in regional or Italian use, and to try to understand the causes.

The species with a reliable attribution absent from the Italian food flora (which does not include cultivated species nor lichens), amount to 34. The category to which the greatest number of absent species is ascribed belongs, predictably, to the less palatable or toxic ones, considered as the last choice as a food resource found in nature. We refer, for example, to *Dryopteris filix-mas*, *Iris Florentina,* and *Paeonia officinalis* which have completely disappeared from the panorama of Italian food species, as well as *Cyclamen hederifolium* and *C. repandum*, whose use has only recently been recorded in Sila, Calabria [111].

The species with a reliable attribution present in the Italian food flora no longer used in Tuscany are 48 (34.29% of the 140 species with a reliable attribution present in the AlimurgITA database and 4.35% of the Italian Alimurgic flora). Regarding Tuscany alone, there is no recent use reported of some species with a decidedly more pleasant taste and that are widely consumed in various Italian regions (e.g., *Celtis australis*, *Tussilago farfara*, *Barbarea vulgaris*, *Eryngium campestre*, *Smyrnium olusatrum*, *Sulla coronaria*). This absence appears at the moment to be inexplicable; in fact, these species are commonly used in regions bordering Tuscany, which boasts a high degree of alimurgical knowledge, the result of numerous thematic articles published (the highest in Italy) that have extensively investigated its entire territory [1]. *Arum italicum* and *A. maculatum* are currently marginal in the gastronomic use of a few Italian regions (Liguria, Veneto, Apulia, Basilicata) and absent in Tuscany. We can also jokingly call them “fallen nobles” because in the past, gigars (especially *Arum italicum*) up to the beginning of the last century enjoyed wide popularity so that their cultivation was encouraged, even with moderate success, thanks to the very high quality and good yields of the starch contained in the rhizomes [19,48,89].

Comparing the species indicated for bread making between the AlimurgITA database and the list of species identified by Targioni Tozzetti, the considerable loss of ethnobotanical knowledge for this specific food use appears with distressing evidence. Among the 198 species reported by Targioni Tozzetti, only 53 are present in Italian alimurgic knowledge nowadays, and unmistakably no longer in popular food practices. The difference becomes even more severe when compared with the data of Tuscany which currently only counts seven species for bread making.

It is interesting to emphasize that this evident erosion of ethnobotanical knowledge (which we can also translate as cultural loss) may be also related to a progressive disappearance of linguistic specificity. This evaluation is based on the comparison between the vernacular terms used in Targioni Tozzetti’s repertoire of plants (342 names) with those reported in the only two synthesis works available on the current ethnobotanical floras of Tuscany [3,41]. In fact, this comparison shows how, as many as 36 names were no longer used (e.g., Alsine, Appio palustre, Iacea, Istia, Been bianco, Linnide saponaria, Mustin greco, Ocimoide bianco, Sio Palustre etc.). Obviously, this datum, in itself quite comforting, could be much more severe if compared with the current knowledge present in the same territories after the disappearance of the generations that still held solidly the culture of popular traditions.

Why were a large number of alimurgical species no longer used in Italy in the last century? The first reason is to be found in the dating of Italian publications on alimurgical topics, which was scarce until the 1970s [1]. Therefore, an important piece of the history of phytoalimurgical narrative is missing, during which ethnobotanical interception occurs when the peasant culture already denotes the progressive and inexorable erosion of its vast and wonderful cultural background.

A second reason lies in the improvement of the general conditions of the population that no longer require the use of plants of poor palatability and for this reason, it is linked to extreme necessity or indigence. This is the period in which, after the 1500s, new edible plants from America appeared; the introduction of which is initially linked to the dramatic famines that forced the European populations to radically review their gastronomic habits. Seen as “alien curiosities” or as livestock fodder, American plants will have acquired the planetary diffusion we know today only after many years [153,154].

For this reason, it seems interesting to note the attention and regret that Giovanni Targioni Tozzetti put on the fact that the cultivation of potatoes and sweet potatoes (*Ipomoea batatas*) wasn’t common in Italy at his time, considering their success in many European countries [6] (p. 10). In fact, it is only since the mid-1800s that, in Italy, the potato (and sometimes sweet potato) has been successfully cultivated and consumed [153] thus providing a considerable contribution towards a richer nutrition for a large part of the population [155]. In the same period, potato has been associated with a rich repertoire of other vegetables (e.g., tomato and bell pepper) which, initially considered of little alimentary value, became with time one of the irreplaceable bases of the Italian diet.

As far as the improvement of general conditions is concerned, the change in climate which signaled the end of the Little Ice Age [156,157,158,159] should not be ignored. The gradual increase in temperature therefore guarantees the possibility of cultivating with greater continuity and profit, largely sheltering from the occurrence of high famines [160].

Nowadays, there is a growing increase in the interest of WEPs in various fields of application that are still a largely unexplored area (e.g., agriculture for a greater availability of highly nutritious food for humanity from sustainable sources, herbal preparations, traditional medicine formulas or new biological drugs [161,162,163,164]).

In light of the above, we believe it is important to give as precise information as possible on toxic food species to which to associate toxicological data in order to give correct information so that the food can be completely safe. And by the way, it should not be forgotten that among the 15 most used alimurgic species, and closely linked to Italian gastronomy spread throughout the country [1], there are some species that are toxic in various capacities (e.g., *Borago officinalis*, *Clematis vitalba* and *Dioscorea communis*).

Furthermore, a range of substances with anti-nutritional action can be found among WEPs. These substances are produced by plants as a defense against herbivores or pathogens (e.g., trypsin inhibitors, phytic acid, tannins, goitrogens, saponins) [165,166,167]. The categories that, for various reasons, must be taken into consideration when choosing the plant products to be included in a diet are diverse. There are many people genetically predisposed to allergic phenomena (e.g., asthma attacks, skin reactions, etc.) or with specific health problems (e.g., kidney diseases aggravated by plant species with high oxalate content).

In the end, the phytoextraction capabilities of many alimurgic plant species should not be underestimated (e.g., *Sonchus oleraceus*, *S. arvensis*, *S. asper*, *Silene vulgaris*, *Lotus corniculatus*, *Salix alba*) [168,169,170,171,172,173]. Their composition in functional compounds can be altered due to the accumulation of toxic substances, thus making plants which were initially considered as good or useful for human health, harmful.

## 5. Conclusions

This work also offered us the opportunity to investigate, from an unusual perspective, a historical period in which the conditions of some social strata, harassed in a state of poverty that was at some times extreme, led to the need and also to the use of ingenuity in finding solutions to feed themselves and to make, as much as possible, the food pleasant. To make, in synthesis, “a virtue of necessity”. In this context, Agostino Pizzorno, editor of the work of Giovanni Targioni Tozzetti, pointed out that the booklet was dictated by a “*sincero sentimento di Umanità per le gravi miserie cagionate alla Toscana*” (sincere feeling of humanity for the grave miseries caused to Tuscany). For this reason, we believe that this writing takes on a human value that goes beyond the scientific one as it places knowledge at the service of human wellbeing.

The comparison of historical botanical data with contemporary data has also given us an opportunity to highlight the continuity or discontinuity of practices linked to popular tradition in specific geographical and cultural contexts.

Looking at the past uses of plants means giving new life to tradition by offering the possibility of their application according to the needs of current communities.

Following in part the path marked out by Giovanni Targioni Tozzetti, we hope that this first contribution on this subject will provide a stimulus to apply this knowledge in the field of modern nutrition, which is often “hungry” for products capable of satisfying particular dietary needs (for example, the use of *Gigaro* or oak acorn flour used as an additive for foods for celiacs).

## Figures and Tables

**Table 1:** The two procedures described by Giovanni Targioni Tozzetti [6] (p. 11) to eliminate toxic substances from the rhizomes and tubers of the eight toxic plants used.

Procedure 1	Procedure 2 (to Be Used as an Alternative to Procedure 1)
Plants: *Felce Maschia* *Felce Femmina* *Asfodelo, Astula Regia, Porreca, Porraccio* *Ciclamino, Panterreno, Panporcino* *Peonia*	Plants: *Pseudacoro, Iride Palustre dal fior giallo* *Iride montana, Giaggiuolo* *Aro, Gichero*
*“Esse ben lavate, pulite, e tagliate in pezzetti, dovranno farsi bollire per un’ora dentro ad una Caldaia con acqua, e con buona dose di Gruma di Botte, o di cenere in un sacchettino; di poi levato il sacchetto, e scolata tutta l’acqua, vi se ne rimetta altra, ma pura, e se gli faccia levare il bollore, e se questa seconda acqua ritenesse ancora del sapore spiacevole delle Radiche, si getti via, e se ne rimetta per la terza volta della pura, e si faccia bollire fintantoché i pezzuoli delle Radiche siano ben cotti e disfatti. Allora si potranno spoltire per impastarne Farina Lievitata di Grano, o di Segale, o d’Orzo o d’Orzuola per uso di Pane…”*	*“Esse Radiche adunque potranno indolcirsi nella maniera poco sopra accennata, ovvero tagliate in pezzetti, si potranno seccare, e macinare, e la loro polvere o Farina si farà bollire per un’ora in una Caldaia con molta acqua, agitandola con un legno, affinché si riduca alla consistenza di Farinata, o di Pulenda fluida; di poi levata la Caldaia dal fuoco, si metta a parte col contenuto per due o tre giorni, e finalmente si decanti, o travasi l’umido che soprannuoterà alla Farina deposta nel fondo, la quale si faccia rasciugare e seccare, per mescolarla con altre Farine, e farne Pane”*
Boil tubers and rhizomes—well washed and cut into pieces—for one hour with *Gruma di Botte* (cream of tartar) or ashes in a bag so that they can be easily removed. Once the bag has been removed, remove the boiling water and, with clean water, bring to the boil again. If necessary, repeat the procedure with clean water a third time until the pieces of tubers and rhizomes are well cooked and dissolved. Once collected, they can be added to leavened wheat, rye, barley or barley flour to make bread.	Dry tubers and rhizomes, reduce them to flour and then boil the powder in plenty of water for an hour, stirring it with a wooden spoon, until it takes on the consistency of a liquid porridge or polenta. Remove the pot from the fire and let the contents decant for two or three days, then remove the liquid part and collect the flour deposited on the bottom. This flour, once dried, can be mixed with other flours in order to make bread.

**Table 2:** List of the 25 species considered harmful by Plants For A Future—PFAF [21]; Species with an indication of toxicity according to Guarrera (*ToxG*) [20] and Acta Plantarum (*ToxAP*) [18]; Species in the database AlimurgITA (*DB*).

Species	ToxG	ToxAP	DB
*Caltha palustris* L.	T	T	No
*Clematis vitalba* L.	T	T	Yes
*Jacobaea vulgaris* Gaertn.	T	T	No
*Saponaria officinalis* L.	T	T	Yes
*Senecio vulgaris* L.	T	T	Yes
*Artemisia vulgaris* L.	T		Yes
*Borago officinalis* L.	T		Yes
*Chenopodium album* L.	T		Yes
*Dryopteris filix-mas* (L.) Schott	T		No
*Oxalis acetosella* L.	T		Yes
*Paeonia officinalis* L.	T		No
*Petroselinum crispum* (Mill.) Fuss	T		Yes
*Rumex acetosa* L.	T		Yes
*Symphytum officinale* L.	T		Yes
*Symphytum tuberosum* subsp. *angustifolium* (A. Kern.) Nyman	T		Yes
*Tussilago farfara* L.	T		Yes
*Verbena officinalis* L.	T		Yes
*Angelica sylvestris* L.		T	Yes
*Ficaria verna* Huds.		T	Yes
*Lathyrus sylvestris* L.		T	Yes
*Limniris pseudacorus* (L.) Fuss		T	No
*Nymphaea alba* L.		T	Yes
*Ornithogalum umbellatum* L.		T	Yes
*Stellaria media* (L.) Vill. subsp. *media*		T	Yes
*Tulipa sylvestris* L.		T	Yes

## Data Availability

The data contained in the AlimurgITA database are available on request from the corresponding author, as the internet portal available to the public is under construction.

## Supplementary Materials

The following are available online at https://www.mdpi.com/article/10.3390/biology11020285/s1, Table S1: List of vernacular names used by Giovanni Targioni Tozzetti accompanied by part used (*PU*), procedure of use (*Procedure*), and toxicity (*Tox*) [6]. It is also reported: if the species is cultivated (*C*), the current name according to the most recent nomenclature for Italy [17] (*Species*), the presence in the database AlimurgITA (*DB*), the indication of toxicity according to Guarrera (*ToxG*) [20], Acta Plantarum (*ToxAP*) [18] and Plants For A Future—PFAF (*Hazard*)—reported only if known and related to the part used [21]; Table S2: List of vernacular names used by Giovanni Targioni Tozzetti [6] for which a match could not be found. Column headings as in Table S1; Table S3: Section A) Taxa in common (37) between list of species from the AlimurgITA database with the one proposed by Giovanni Targioni Tozzetti; Section B) taxa NOT in common (16) between the list of species from the AlimurgITA database with the one proposed by Giovanni Targioni Tozzetti.
