# Supplementary material for: Making a Virtue of Necessity: The Use of Wild Edible Plant Species (Also Toxic) in Bread Making in Times of Famine According to Giovanni Targioni Tozzetti (1766)
Source: Biology (Basel). 2022 Feb 11;11(2):285. doi: 10.3390/biology11020285 (PMC8869735; doi:10.3390/biology11020285)
Supplement: Supplementary file 1 [file biology-11-00285-s001.zip › biology-1539382-supplementary.pdf]

Table S1. List of vernacular names used by Giovanni Targioni Tozzetti accompanied by part used (*PU*), procedure of use (*Procedure*), and toxicity (*Tox*) [6]. It is also reported: if the species is cultivated (*C*), the current name according to the most recent nomenclature for Italy [17] (*Species*), the presence in the AlimurgITA database (*DB*), the indication of toxicity according to Guarrera (*ToxG*) [20], Acta Plantarum (*ToxAP*) [18] and Plants For A Future - PFAF (*Hazard* - reported only if known and related to the part used) [21].

Vernacular name	PU	Procedure	Tox	C	Species	Family	DB	Tox G	Tox AP	Hazard
Abeti	Seeds	Dried seeds are ground in order to obtain flour to be mixed with bread dough			*Abies alba* Miller	Pinaceae	Yes			
					Picea abies (L.) H. Karst.		Yes			
Acanto o Branca Orsina	Leaves and tops	Raw leaves and tops are crushed and added to bread dough			*Acanthus mollis* L.	Acanthaceae	No			
Acero	Seeds	The seeds, bitter, must be boiled with a double change of water and then left three days in running water. Once dried, they are ground in order to obtain flour to be mixed to bread dough			*Acer platanoide*s L.	Sapindaceae	No			
					Acer pseudoplatanus L.		Yes			
Acero detto Loppo	Tips or tops and tender twigs	Tips, tops or tender twigs must be cooked before adding them to bread dough			*Acer campestre* L.		Yes			
					Acer opalus Mill.		Yes			
Acetosa salvatica	Leaves	The leaves can be used to give a pretty sour flavour to unflavoured or too sweet bread doughs; they are useful against scurvy			*Rumex acetosa* L.	Polygonaceae	Yes	T		It contains high levels of oxalic acid, the quantity of which can be reduced by cooking.
Aglio	Roots	The bulbs can be used to modify the bitter and sour taste of some acorns		C	*Allium sativum L.*	Amaryllidaceae	No			
Agrimonia	Leaves	The leaves can be used to give a pretty sour flavour to unflavoured or too sweet bread doughs; they are useful against scurvy			*Agrimonia eupatoria* L.	Rosaceae	Yes			
Albicocche	Fruits	Fruits, dried in segments, can be crushed and mixed to bread dough; alternatively fresh or dried segments can be cooked first and then added to bread dough		C	*Prunus armeniaca* L.		No			
Alcea	Leaves	Leaves are boiled, if necessary twice with water change, then pounded, sieved and added to bread dough			*Althaea cannabina* L.	Malvaceae	No			
Alchimilla	Tips or tops and tender twigs	Tips, tops or tender twigs must be cooked before adding them to bread dough			*Alchemilla vulgaris* L.	Rosaceae	Yes			
Alimo	Leaves	Leaves are boiled, if necessary twice with water change, then pounded, sieved and added to bread dough			*Atriplex halimus* L.	Amaranthaceae	Yes			Leaves can concentrate harmful amounts of nitrates if the plant grows on nitrogen-rich soils

Alloro Coccole di Aloro	Fruits<br><br>Leaves	The dried fruits are broken to extract the seed, which is boiled with ash or barrel lump and then proceeded as for *Lupinus albus*<br>The leaves can be used to modify the bitter and sour taste of some acorns			*Laurus nobilis* L.	Lauraceae	Yes			
Alsine detta Centonchio	Leaves and tops	Raw leaves and tops are crushed and added to bread dough			*Stellaria media* (L.) Vill. subsp. *media*	Caryophyllaceae	Yes		T	The leaves contain saponins, which are very poorly absorbed by the body and are also broken down by thorough cooking.
Altea	Leaves	Leaves are boiled, if necessary twice with water change, then pounded, sieved and added to bread dough			*Althaea officinalis* L.	Malvaceae	Yes			
Amaranti	Leaves	Leaves are boiled, if necessary twice with water change, then pounded, sieved and added to bread dough			*Amaranthus hybridus* subsp. *cruentus* (L.) Thell.	Amaranthaceae	Yes			Leaves can concentrate harmful amounts of nitrates if the plant grows on nitrogen-rich soils
Ambrostoli, Uve salvatiche, uva Rampina, Vite salvatica	Fruits<br><br>Tender shoots and vines<br><br>Flowers<br><br>Tendrils	Small fruits, dried, can be added directly to bread dough<br>Cooked tender shoots and vines can be added to bread, but it would be better to eat them alone<br>Dried and pulverized flowers can be added to bread dough to improve the flavour of bland, cloying or flatulent mixtures<br>The young tendrils can be used to give a pretty sour flavour to unflavoured or too sweet bread doughs; they are useful against scurvy		C	*Vitis labrusca* L.	Vitaceae	No			
Anacio	Seeds	Boil seeds with legumes, or use their decoction to knead bread that contains legume flour, to avoid mixtures that may cause flatulence			*Pimpinella anisum* L.		No			
Angelica Montana	Leaves and Seeds	Dried and pulverized leaves and seeds, can be added to bread dough to improve the flavour of bland, cloying or flatulent mixtures			*Angelica archangelica* L.	Apiaceae	No			All members of this genus contain furocoumarins, which increase skin sensitivity to sunlight and may cause dermatitis
Angelica Silvestre	Leaves and Seeds	Dried and pulverized leaves and seeds, can be added to bread dough to improve the flavour of bland, cloying or flatulent mixtures			*Angelica sylvestris* L.		Yes		T	All members of this genus contain furocoumarins, which increase skin sensitivity to sunlight and may cause dermatitis
Anonide detta Bulimacola	Young sprouts	Cooked young sprouts can be added to bread, but it would be better to eat them alone			*Ononis spinosa* subsp. *arvensis* (L.) Greuter & Burdet	Fabaceae	Yes			
Appio Palustre	Leaves and Seeds	Dried and pulverized leaves and seeds, can be added to bread dough to improve the flavour of bland, cloying or flatulent mixtures		C	*Apium graveolens* L.	Apiaceae	Yes			Cross-allergenicity between celery, cucumber, carrot, watermelon and possibly apples. Avoid during pregnancy as emmenagogue, abortifacient and uterine stimulant activity reported
Aro o Gichero	Roots	The procedure is described in Table 1	T		*Arum italicum* Mill.	Araceae	Yes	T	T	

			T		*Arum maculatum* L.		Yes	T	T	The plant contains calcium oxylate crystals. These cause an extremely unpleasant sensation similar to needles being stuck into the mouth and tongue if they are eaten, but they are easily neutralized by thoroughly drying or cooking the plant or by steeping it in water
Artemisia	Tips or tops and tender twigs	Tips, tops or tender twigs must be cooked before adding them to bread dough			*Artemisia vulgaris* L.	Asteraceae	Yes	T		It might be poisonous in large doses. Probably unsafe for pregnant women as it may induce abortion
Asfodelo, Astula regia	Roots	The procedure is described in Table 1	T		*Asphodeline lutea* (L.) Rchb.	Asphodelaceae	Yes		T	
			T		*Asphodelus ramosus* L.		Yes			
Aster detto Tripolio	Tips or tops and tender twigs	Tips, tops or tender twigs must be cooked before adding them to bread dough			*Galatella tripolium* (L.) Galasso, Bartolucci & Ardenghi	Asteraceae	No			
Astragalo	Tips or tops and tender twigs	Tips, tops or tender twigs must be cooked before adding them to bread dough			*Astragalus cicer* L.	Fabaceae	No			Many members of this genus contain toxic glycosides. A number of species can also accumulate toxic levels of selenium when grown in soils that are relatively rich in that element
					Astragalus exscapus L.		No			
					Astragalus glycyphyllos L.		Yes			
					Astragalus hamosus L.		Yes			
					Astragalus sesameus L.		No			
Atriplici salvatici	Leaves	Leaves are boiled, if necessary twice with water change, then pounded, sieved and added to bread dough			*Chenopodium album* L.	Amaranthaceae	Yes	T		The leaves contain saponins, which are very poorly absorbed by the body and are also broken down by thorough cooking.<br>The plants also contain some oxalic acid, which in large quantities can lock up some of the nutrients in the food, but these plants are very nutritious vegetables in reasonable quantities. Cooking the plant will reduce its content of oxalic acid, and then only if large quantities are consumed.<br>Leaves can concentrate harmful amounts of nitrates if the plant grows on nitrogen-rich soils. In nitrogen-rich soils, the plants can also concentrate hydrogen cyanide. In small quantities, hydrogen cyanide has been shown to stimulate respiration and improve digestion, it is also claimed to be of benefit in the treatment of cancer.
Avornello	Seeds	The seeds, bitter, must be boiled with a double change of water and then left three days in running water. Once dried, they are ground in order to obtain flour to be mixed to bread dough			*Fraxinus ornus* L. subsp. *ornus*	Oleaceae	Yes			

Bagole, cioé frutti di vite idea o Mirtillo	Fruits	Sour fruits, fresh or dried, are cooked in water, sieved and then added to bread dough. In alternative the small fruits, dried, can be added directly to bread dough			*Vaccinium myrtillus* L.	Ericaceae	Yes			High tannin content may cause digestive disorders
					Vaccinium vitis-idea L.		Yes			It contains the toxin “arbutin”
Barbarea	Leaves	Leaves are useful against scurvy			*Barbarea vulgaris* W.T. Aiton	Brassicaceae	Yes			There is a report that ingestion of the leaves can lead to kidney malfunction
Batate	Roots	There is only one indication for a desirable, but not yet possible use		C	*Ipomoea batatas* (L.) Lam.	Convolvulaceae	No			
Beccabunga	Leaves and tops	Raw leaves and tops are crushed and added to bread dough			*Veronica anagallis-aquatica* L.	Plantaginaceae	Yes			
					Veronica beccabunga L.		Yes			
Been bianco	Tips or tops and tender twigs	Tips, tops or tender twigs must be cooked before adding them to bread dough			*Silene vulgaris* (Moench) Garcke	Caryophyllaceae	Yes			The plant contain saponins, which are very poorly absorbed by the body and are also broken down by thorough cooking.
Bellide o Fior di Prato	Leaves	Leaves are boiled, if necessary twice with water change, then pounded, sieved and added to bread dough			*Bellis perennis* L.	Asteraceae	Yes			
Berberi o Crespino	Fruits	Sour fruits, fresh or dried, are cooked in water, sieved and then added to bread dough. In alternative the small fruits, dried, can be added directly to bread dough			*Berberis vulgaris* L.	Berberidaceae	Yes			
Bietola, Barbe Bietole	Roots	Roots, dried in pieces, can be crushed and mixed to bread dough. Alternatively, fresh pieces can be cooked, sieved and then added to bread dough			*Beta vulgaris* L.	Amaranthaceae	Yes			
Biodi, Biodri	Seeds<br>Roots	Dried seeds are ground in order to obtain flour to be mixed with bread dough<br>Young roots, fresh or boiled, are crushed and added to bread dough			*Butomus umbellatus* L.	Butomaceae	Yes			
					Sparganium erectum L.	Typhaceae	Yes			
					Schoenplectus lacustris (L.) Palla	Cyperaceae	Yes			
					Typha latifolia L.	Thyphaceae	Yes			
					Typha angustifolia L.		Yes			
Bislingua	Young sprouts	Cooked young sprouts can be added to bread, but it would be better to eat them alone			*Ruscus hypophyllum* L.	Asparagaceae	Yes			
Bistorta	Roots<br>Leaves	The dried roots are crushed and sieved and the resulting powder is boiled, then decanted and the water is thrown away to add new water and boiled a second time, then the powder is added to the bread dough<br>Leaves are boiled, if necessary twice with water change, then pounded, sieved and added to bread dough			*Bistorta officinalis* Delarbre	Polygonaceae	Yes			Many species contain oxalic acid, the quantity of which can be reduced by cooking.

Bliti	Leaves	Leaves are boiled, if necessary twice with water change, then pounded, sieved and added to bread dough			*Amaranthus blitum* L.	Amaranthaceae	Yes			Leaves can concentrate harmful amounts of nitrates if the plant grows on nitrogen-rich soils.
					Amaranthus hybridus subsp. *caudatus* (L.) Iamonico & Galasso		Yes			
Borrana	Leaves and tops	Raw leaves and tops are crushed and added to bread dough			*Borago officinalis* L.	Boraginaceae	Yes	T		The plant contains small amounts of pyrrolizidine alkaloids that can cause liver damage and liver cancer. These alkaloids are present in too small a quantity to be harmful unless you make borage a major part of your diet
Brunella	Tips or tops and tender twigs	Tips, tops or tender twigs must be cooked before adding them to bread dough			*Prunella vulgaris* L.	Lamiaceae	Yes			
Buglossa	Leaves and tops	Raw leaves and tops are crushed and added to bread dough			*Anchusa azurea* Mill.	Boraginaceae	Yes			
					Anchusa officinalis L.		Yes			
Bursa Pastoris	Leaves	Leaves are useful against scurvy			*Capsella bursa-pastoris* (L.) Medik.	Brassicaceae	Yes			
Calamento, Calamento montano	Leaves<br><br>Leaves and tops	Boil leaves with legumes, or use their decoction to knead bread that contains legume flour, to avoid mixtures that may cause flatulence<br>Dried and pulverized leaves and tops, can be added to bread dough to improve the flavour of bland, cloying or flatulent mixtures			*Clinopodium nepeta* subsp. *spruneri* (Boiss.) Bartolucci & F. Conti	Lamiaceae	No			
Calcitrapa	Tips or tops and tender twigs	Tips, tops or tender twigs must be cooked before adding them to bread dough			*Centaurea calcitrapa* L.	Asteraceae	Yes			
Calta o Calendula o Fior Rancio	Tips or tops and tender twigs	Tips, tops or tender twigs must be cooked before adding them to bread dough			*Calendula officinalis* L.		Yes			Low potential for sensitization and contact dermatitis. Possible allergies if allergic to daisy family plants.
Camenerio	Young sprouts	Cooked young sprouts can be added to bread, but it would be better to eat them alone			*Chamaenerion angustifolium* (L.) Scop.	Onagraceae	Yes			An infusion of the leaves is said to stupefy a person
Campanule	Leaves	Leaves are boiled, if necessary twice with water change, then pounded, sieved and added to bread dough			*Campanula persicifolia* L.	Campanulaceae	Yes			
					Campanula medium L.		Yes			
Canapa	Seeds	Dried seeds are ground in order to obtain flour to be mixed with bread dough as long as in small quantity		C	*Cannabis sativa* L.	Cannabaceae	No			It is a narcotic
Canne	Roots	The roots, fresh or boiled, are crushed and added to bread dough			*Arundo donax* L.	Poaceae	Yes			
					Phragmites australis (Cav.) Trin. ex Steud.		Yes			

Cardi o Scardiccioni	Seeds<br><br>Roots<br><br>Tips or tops and tender twigs	Seeds crushed and pressed in water produce an aromatic water that serves as a flavouring agent for baking flours with a sour or bitter taste<br><br>The roots, boiled a first time with a small bag of ashes and a second time with pure water, are sieved and added to the bread dough<br><br>Tips, tops or tender twigs must be cooked before adding them to bread dough			*Carduus nutans L.*	Asteraceae	Yes			
					Carduus pycnocephalus L.		Yes			
					Carthamus lanatus L.		Yes			
					Centaurea solstitialis L.		Yes			
					Cirsium arvense (L.) Scop.		Yes			
					Cirsium oleraceum (L.) Scop.		Yes			
					Cynara cardunculus subsp. *scolymus* (L.) Hegi	See ***Scolimo***				
					Dipsacus fullonum L.	See ***Dissaco o Labrum Veneris o Scardaccio***				
					Drypis spinosa L.	Caryophyllaceae	No			
					Echinops sphaerocephalus L. subsp. *sphaerocephalus*	Asteraceae	No			
					Echinops strigosus L.		No			
					Genista germanica L.	Fabaceae	No			
					Onopordum acanthium L	See ***Onopordo o Acanzio***				
					Ptilostemon stellatus (L.) Greuter	Asteraceae	No			
					Scolymus hispanicus L.		Yes			
					Scolymus maculatus L.		Yes			
					Silybum marianum (L.) Gaertn.		Yes			Leaves can concentrate harmful amounts of nitrates if the plant grows on nitrogen-rich soils
Carici	Seeds	Dried seeds are ground in order to obtain flour to be mixed with bread dough			*Carex acuta* L.	Cyperaceae	No			
					Carex nigra (L.) Reichard		No			
Carlina	Leaves	Leaves are boiled, if necessary twice with water change, then pounded, sieved and added to bread dough			*Carlina acaulis* L.	Asteraceae	Yes			
Castagne<br><br>***Castagno***	Seeds<br><br>Tips or tops and tender twigs	Dried seeds, once peeled, are ground in order to obtain flour to be mixed with bread dough<br>Tips, tops or tender twigs must be cooked before adding them to bread dough			*Castanea sativa* Mill.	Fagaceae	Yes			
Ceci	Seeds	The seeds are boiled to make a mush to be mixed with the bread dough		C	*Cicer arietinum* L.	Fabaceae	No			

Cerro	Seeds	Dried seeds are ground in order to obtain flour. Before using it, acorn flour must be boiled until it becomes a liquid paste, then it is added to bread dough			*Quercus cerris* L.	Fagaceae	Yes			
Cetriuoli	Fruits	Boiled fruit pulp is added to bread dough		C	*Cucumis sativus* L.	Cucurbitaceae	No			
Chenopodj	Leaves	Leaves are boiled, if necessary twice with water change, then pounded, sieved and added to bread dough			*Chenopodium album* L.	See ***Atriplici salvatici***				
					Dysphania ambrosioides (L.) Mosyakin & Clemants	Amaranthaceae	Yes			
Cicerchie	Seeds<br>Leaves and tops	The seeds are boiled to make a mush to be mixed with the bread dough<br>Raw leaves and tops are crushed and added to bread dough			*Lathyrus sativus* L.	Fabaceae	Yes			The seed contains a toxic amino-acid which can cause 'lathyrism' (a disease of the nervous system) if ingested in large quantities.
Ciclamino detto Panterreno, Panporcino	Roots	The procedure is described in table 1	T		*Cyclamen hederifolium* Aiton	Primulaceae	Yes	T	T	It is poisonous
			T		*Cyclamen repandum* Sm. subsp. *repandum*		Yes	T	T	
Cimbalaria	Leaves	Leaves are boiled, if necessary twice with water change, then pounded, sieved and added to bread dough			*Cymbalaria muralis* G.Gaertn., B.Mey. & Scherb.	Plantaginaceae	Yes			It might be slightly toxic
Cipperi	Seeds<br>Roots	Dried seeds are ground in order to obtain flour to be mixed with bread dough;			*Cyperus longus* L.	Cyperaceae	Yes			
					Cyperus rotundus L.		Yes			
Cirsj	Tips or tops and tender twigs	Tips, tops or tender twigs must be cooked before adding them to bread dough			*Cirsium vulgare* (Savi) Ten.	Asteraceae	Yes			
Citisi	Tips or tops and tender twigs	Tips, tops or tender twigs must be cooked before adding them to bread dough			*Medicago arborea* L.	Fabaceae	No			
Clematide o Vitalba	Tender shoots and vines	Cooked tender shoots and vines can be added to bread, but it would be better to eat them alone			*Clematis vitalba* L.	Ranunculaceae	Yes	T	T	It is poisonous. The toxic principle, present in less quantity in young parts, is dissipated by heat or by drying
Climeno	Seeds	The seeds are boiled to make a mush to be mixed with the bread dough			*Lathyrus clymenum* L.	Fabaceae	Yes			
Cocomeri	Tender shoots and vines	Cooked tender shoots and vines can be added to bread, but it would be better to eat them alone		C	*Citrullus lanatus* (Thunb.) Matsum. & Nakai	Cucurbitaceae	No			
Corbezzole	Fruits	Sour fruits, fresh or dried, are cooked in water, sieved and then added to bread dough			*Arbutus unedo* L.	Ericaceae	Yes			
Corniole	Fruits	Sour fruits, fresh or dried, are cooked in water, sieved and then added to bread dough			*Cornus mas* L.	Cornaceae	Yes			

Coronopo o Erba stella	Leaves	Leaves are boiled, if necessary twice with water change, then pounded, sieved and added to bread dough			*Plantago coronopus* L.	Plantaginaceae	Yes			
Crimmo detto Finocchio Marino, Bacicci, o Erba San Pietro	Leaves and Seeds	Dried and pulverized leaves and seeds, can be added to bread dough to improve the flavour of bland, cloying or flatulent mixtures			*Crithmum maritimum* L.	Apiaceae	Yes			
Cruciata	Tips or tops and tender twigs	Tips, tops or tender twigs must be cooked before adding them to bread dough			*Cruciata glabra* (L.) C. Bauhin ex Opiz	Rubiaceae	No			
Dente di Leone	Leaves and tops	Raw leaves and tops are crushed and added to bread dough			*Taraxacum officinale* Weber s.l.	Asteraceae	Yes			
Dissaco o Labrum Veneris o Scardaccio	Leaves	Leaves are boiled, if necessary twice with water change, then pounded, sieved and added to bread dough			*Dipsacus fullonum* L.	Caprifoliaceae	Yes			
Draba	Leaves	Leaves are useful against scurvy			*Drabella muralis* (L.) Fourr.	Brassicaceae	No			
Echio	Leaves	Leaves are boiled, if necessary twice with water change, then pounded, sieved and added to bread dough			*Echium italicum* L.	Boraginaceae	Yes	T		
					Echium vulgare L.		Yes	T		Leaves are poisonous
Edisaro detto Lupinello	Tips or tops and tender twigs	Tips, tops or tender twigs must be cooked before adding them to bread dough			*Sulla coronaria* (L.) B.H.Choi & H.Ohashi	Fabaceae	Yes			
Emionitide	Leaves<br><br>Tips or tops and tender twigs	Leaves are boiled, if necessary twice with water change, then pounded, sieved and added to bread dough<br>Tips, tops or tender twigs must be cooked before adding them to bread dough			*Asplenium sagittatum* (DC.) Bange	Aspleniaceae	No			
Equiseto detto Coda di Cavallo	Young sprouts	Cooked young sprouts can be added to bread, but it would be better to eat them alone			*Equisetum arvense* L.	Equisetaceae	Yes			It contains *thiaminase*, an enzyme that robs the body of its vitamin B complex, which is destroyed by heat or thorough drying<br><br>The plant also contains equisetic acid.
					Equisetum hyemale L.		No			
					Equisetum sylvaticum L.		No			
Erba Kali	Leaves and tops	Finely crushed and sifted, are used instead of salt to flavour bread doughs			*Salsola kali* L.	Amaranthaceae	No			It contain up to 5% oxalic acid, the quantity of which can be reduced by cooking.
					Salsola tragus L.		No			
					Soda inermis Fourr.	Chenopodiaceae	Yes			
Eringio o Calcatreppola	Roots	The roots, boiled a first time with a small bag of ashes and a second time with pure water, are sieved and added to the bread dough			*Eryngium campestre* L.	Apiaceae	Yes			

Erucaggine detta Cascellore	Leaves	Leaves are boiled, if necessary twice with water change, then pounded, sieved and added to bread dough			*Bunias erucago* L.	Brassicaceae	Yes			
Eupatorio Cannabino	Tips or tops and tender twigs	Tips, tops or tender twigs must be cooked before adding them to bread dough			*Eupatorium cannabinum* L.	Asteraceae	Yes		T	
Faggiuole<br><br>***Faggio***	Seeds<br><br>Tips or tops and tender twigs	Dried seeds, once peeled, are ground in order to obtain flour to be mixed with bread dough<br>Tips, tops or tender twigs must be cooked before adding them to bread dough			*Fagus sylvatica* L.	Fagaceae	Yes			Seeds may be toxic in large quantities
Fagiuoli	Seeds	The seeds are boiled to make a mush to be mixed with the bread dough		C	*Phaseolus vulgaris* L.	Fabaceae	No			Raw mature seed are poisonous in large quantities
Farfarugio	Leaves	Leaves are boiled, if necessary twice with water change, then pounded, sieved and added to bread dough			*Caltha palustris* L.	Ranunculaceae	No	T	T	The whole plant, but especially the older portions, contains the toxic glycoside *protoanemonin* which is destroyed by heat.
					Tussilago farfara L.	See ***Tosillaggine o Farfero***				
Farnia	Tips or tops and tender twigs	Tips, tops or tender twigs must be cooked before adding them to bread dough			*Quercus robur* L.	Fagaceae	Yes			Possible digestive complaints.
Favagello	Roots	The roots, boiled to one water and sieved can be added to the bread dough			*Ficaria verna* Huds.	Ranunculaceae	Yes		T	It is poisonous. The toxins, unstable and of low toxicity, are destroyed by heat or drying.
Fave	Seeds<br><br>Leaves and tops	The seeds are boiled to make a mush to be mixed with the bread dough<br>Raw leaves and tops are crushed and added to bread dough		C	*Vicia faba* L.	Fabaceae	No			Although often used as an edible seed, there is a report that eating the seed of this plant can cause the disease 'Favism' in susceptible people.
Felce femmina	Roots	The procedure is described in Table 1	T		*Athyrium filix-femina* (L.) Roth	Pteridaceae	Yes			Although we have found no reports for this species, a number of ferns also contain carcinogens
Felce maschia	Roots	The procedure is described in Table 1	T		*Dryopteris filix-mas* (L.) Schott	Dryopteridaceae	No	T		Although we have found no reports for this species, a number of ferns contain carcinogens
Filipendula	Roots	The dried roots are crushed and sieved and the resulting powder is boiled, then decanted and the water is thrown away to add new water and boiled a second time, then the powder is added to the bread dough			*Filipendula ulmaria* (L.) Maxim.	Rosaceae	Yes			Avoid for asthmatics. Use by children for diarrhoea not recommended or for children under 12 due to salicylate content (risk of Reye's syndrome)
					Oenanthe pimpinelloides L.	Apiaceae	Yes			Many species in this genus are very poisonous, one report has been found so far saying that this species is also poisonous. It is said to contain the alleged 'psychotroph' myristicine
Finocchio	Leaves and Seeds	Boil seeds with legumes, or use their decoction to knead bread that contains legume flour, to avoid mixtures that may cause flatulence			*Foeniculum vulgare* Mill.		Yes	T		

		Dried and pulverized leaves and seeds, can be added to bread dough to improve the flavour of bland, cloying or flatulent mixtures								
Formentone, Grano Siciliano o Turco	Seeds	Dried seeds are ground into bread flour		C	*Zea mays* L.	Poaceae	No			
Fragole	Leaves	Leaves are boiled, if necessary twice with water change, then pounded, sieved and added to bread dough			*Fragaria vesca* L.	Rosaceae	Yes			
Frassino	Seeds	The seeds, bitter, must be boiled with a double change of water and then left three days in running water. Once dried, they are ground in order to obtain flour to be mixed to bread dough			*Fraxinus excelsior* L.	Oleaceae	No			
Fuchi marini		Finely crushed and sifted, are used instead of salt to flavour bread doughs			*Sargassum natans* (L.) Gaillon	Sargassaceae	No			
					Fucus vesiculosus L.	Fucaceae	No			
Galega detta Capraggine	Tips or tops and tender twigs	Tips, tops or tender twigs must be cooked before adding them to bread dough			*Galega officinalis* L.	Fabaceae	Yes			A few reports exist of toxicity to mammals
Girasole o Pianta Massima	Seeds	Seeds crushed and pressed in water produce an aromatic water that serves as a flavouring agent for baking flours with a sour or bitter taste		C	*Helianthus annuus* L.	Asteraceae	No			
Gramigne	Roots	The roots, fresh or boiled, are crushed and added to bread dough			*Cynodon dactylon* (L.) Pers.	Poaceae	Yes			
	Leaves and tops	Raw leaves and tops are crushed and added to bread dough			*Elymus repens* (L.) Gould subsp. *repens*		Yes			Prolonged use may lead to loss of potassium due to its diuretic action
Gran Farro	Seeds	Dried seeds are ground into bread flour		C	*Triticum aestivum* subsp. *spelta* (L.) Thell.		No			
Grano duro	Seeds	Dried seeds are ground into bread flour		C	*Triticum turgidum* subsp. *durum* (Desf.) Husn.		No			
Grano dolce sì Gentile, che Grosso	Seeds	Dried seeds are ground into bread flour		C	*Triticum aestivum* L.		No			
Iacea	Tips or tops and tender twigs	Tips, tops or tender twigs must be cooked before adding them to bread dough			*Viola tricolor* L.	Violaceae	Yes			
Iacobea	Tips or tops and tender twigs	Tips, tops or tender twigs must be cooked before adding them to bread dough			*Jacobaea vulgaris* Gaertn.	Asteraceae	No	T	T	It is poisonous (pyrrolizidine alkaloids)
Iberide	Leaves	Leaves are useful against scurvy			*Lepidium graminifolium* L.	Brassicaceae	Yes			

Ieracj di sapor dolce <br>***Ieraci amarognoli***	Leaves and tops (sweet or bland ones) <br>Leaves (bitter ones)	Raw leaves and tops are crushed and added to bread dough Leaves are boiled, if necessary twice with water change, then pounded, sieved and added to bread dough			*Pilosella officinarum* Vaill.	Asteraceae	Yes			
					Hieracium sabaudum L.		No			
					Hieracium umbellatum L.		No			
Iride montana o Giaggiuolo	Roots	The procedure is described in Table 1	T		*Iris florentina* L.	Iridaceae	No			The rhizomes contain an irritating resinous substance called *irisin*. If ingested this can cause severe gastric disturbances.
			T		*Iris germanica* L.		Yes			
Istia	Tips or tops and tender twigs	Tips, tops or tender twigs must be cooked before adding them to bread dough			*Phillyrea latifolia* L.	Oleaceae	No			
Lampone	Fruits	Small fruits, dried, can be added directly to bread dough			*Rubus idaeus* L.	Rosaceae	Yes			
Lapato	Roots <br>Leaves	The dried roots are crushed and sieved and the resulting powder is boiled, then decanted and the water is thrown away to add new water and boiled a second time, then the powder is added to the bread dough Leaves are boiled, if necessary twice with water change, then pounded, sieved and added to bread dough			*Rumex conglomeratus* Murray	Polygonaceae	Yes			Leaves can contain high levels of oxalic acid, the quantity of which can be reduced by cooking.
					Rumex sanguineus L.		Yes			
Leccio	Seeds	Dried seeds are ground in order to obtain flour. Before using it, acorn flour must be boiled until it becomes a liquid paste, then it is added to bread dough			*Quercus ilex* L. subsp. *ilex*	Fagaceae	Yes			
Lenti	Seeds	The seeds are boiled to make a mush to be mixed with the bread dough		C	*Vicia lens* (L.) Coss. & Germ.	Fabaceae	No			
Lenticchia Palustre	Leaves and tops	Raw leaves and tops are crushed and added to bread dough			*Lemna minor* L.	Araceae	No			
					Spirodela polyrhiza (L.) Schleid.		No			
Lentisco	Seeds	Seeds crushed and pressed in water produce an aromatic water that serves as a flavouring agent for baking flours with a sour or bitter taste			*Pistacia lentiscus* L.	Anacardiaceae	Yes			Small risk of diarrhoea in children. Avoid oral intake of essential oil
Lepidio	Leaves	Leaves are useful against scurvy			*Lepidium latifolium* L.	Brassicaceae	Yes			
Licheni	Thallus	Lichen dried and reduced in powder, can be added to bread dough			*Cetraria islandica* (L.) Ach.	Parmeliaceae	No			Indigestion and nausea with large doses. Rare liver damage.
Lingua Cervina	Leaves	Leaves are boiled, if necessary twice with water change, then pounded, sieved and added to bread dough			*Asplenium scolopendrium* L.	Aspleniaceae	No			Although we have found no reports of toxicity for this species, a number of ferns contain carcinogens.

	Tips or tops and tender twigs	Tips, tops or tender twigs must be cooked before adding them to bread dough								Many ferns contain *thiaminase*, an enzyme that robs the body of its vitamin B complex, which is destroyed by heat or thorough drying
Linnide	Tips or tops and tender twigs	Tips, tops or tender twigs must be cooked before adding them to bread dough twigs			*Lychnis coronaria* (L.) Desr.	Caryophyllaceae	No			
					Silene dioica (L.) Clairv.		Yes			The plant contain saponins, which are very poorly absorbed by the body and are also broken down by thorough cooking.
Linnide saponaria Erba saponaria	Leaves and tops	Raw leaves and tops are crushed and added to bread dough			*Saponaria officinalis* L.		Yes	T	T	The leaves contain saponins, which are very poorly absorbed by the body and are also broken down by thorough cooking.
Lino	Seeds	Dried seeds are ground in order to obtain flour to be mixed with bread dough as long as in small quantity		C	*Linum usitatissimum* L.	Linaceae	Yes			The seed can contain cyanogenic glycosides
Loto Pratense	Tips or tops and tender twigs	Tips, tops or tender twigs must be cooked before adding them to bread dough			*Lotus maritimus*	Fabaceae	Yes			
Loto detto Fraggiragolo, o Bagatto, o Bucerata	Fruits	Sour fruits, fresh or dried, are cooked in water, sieved and then added to bread dough			*Celtis australis* L. subsp. *australis*	Ulmaceae	Yes			
Lunaria	Tips or tops and tender twigs	Tips, tops or tender twigs must be cooked before adding them to bread dough			*Lunaria annua* L.	Brassicaceae	Yes			
Lupini	Seeds	The seeds, bitter, must be boiled with a double change of water and then left three days in running water. Once dried, the shell is removed and they are ground in order to obtain flour to be mixed to bread dough			*Lupinus albus* L.	Fabaceae	Yes	T		The seed of many species of this genus contain bitter-tasting toxic alkaloids. These alkaloids can be leeched out of the seed by soaking it overnight and discarding the soak water, but it may also be necessary to change the water once during cooking.
Luppolo	Young sprouts	Cooked young sprouts can be added to bread, but it would be better to eat them alone			*Humulus lupulus* L.	Cannabaceae	Yes			Skin contact with the plant causes dermatitis in sensitive people. Dislodged hairs from the plant can irritate the eyes. Sedative effect may worsen depression. Avoid during pregnancy (due to antispasmodic action on uterus).
Macerone	Tips or tops and tender twigs	Tips, tops or tender twigs must be cooked before adding them to bread dough			*Smyrnium olusatrum* L.	Apiaceae	Yes			
Malva	Leaves	Leaves are boiled, if necessary twice with water change, then pounded, sieved and added to bread dough			*Malva neglecta* Wallr.	Malvaceae	Yes			Leaves can concentrate harmful amounts of nitrates if the plant grows on nitrogen-rich soils
					Malva sylvestris L.		Yes			
Medica	Leaves and tops	Raw leaves and tops are crushed and added to bread dough			*Medicago sativa* L.	Fabaceae	Yes			The leaves contain *saponins*-like substances, which are very poorly absorbed by the body and

										are also broken down by thorough cooking (and perhaps changing the cooking water once).<br><br>The sprouts (and especially the seeds) contain *canavanine*, which can cause the recurrence *of* systemic lupus erythematosus in patients where the disease had become dormant.
Melampiro o Grano Vaccino o Bovino o Nero	Seeds	Dried seeds are ground in order to obtain flour to be mixed with bread dough			*Melampyrum arvense* L	Orobanchaceae	No		T	
Mele<br><br>***Melo***	Fruits<br><br>Tips or tops and tender twigs	Sweet fruits, dried in segments, can be crushed and mixed to bread dough; alternatively, fresh or dried segments can be cooked first and then added to bread dough; sour fruits, fresh or dried, are cooked in water, sieved and then added to bread dough Tips, tops or tender twigs must be cooked before adding them to bread dough		C	*Malus domestica* (Suckow) Borkh.	Rosaceae	Yes			
Melissa detta Cedrencella	Leaves and tops	Dried and pulverized leaves and tops, can be added to bread dough to improve the flavour of bland, cloying or flatulent mixtures			*Melissa officinalis* L.		Yes			Can cause irritation in high concentrates. Avoid during pregnancy. Care if sensitive skin
Menta	Leaves and tops	Dried and pulverized leaves and tops, can be added to bread dough to improve the flavour of bland, cloying or flatulent mixtures			*Mentha spicata* L.	Lamiaceae	Yes			In large quantities it can cause abortions
					Mentha x *piperita* L.		Yes			
Mentastro	Leaves and tops	Dried and pulverized leaves and tops, can be added to bread dough to improve the flavour of bland, cloying or flatulent mixtures			*Mentha longifolia* (L.) L.		Yes			Large quantities of some members of this genus can cause abortions
					Mentha suaveolens Ehrh.		Yes			
Miglio, Panico	Seeds	Dried seeds are ground into bread flour		C	*Panicum miliaceum* L.	Poaceae	Yes			
Mocaione	Seeds	The seeds are boiled to make a mush to be mixed with the bread dough			*Lathyrus latifolius* L.	Fabaceae	Yes			Although no records of toxicity have been found for this plant, the seed of some species in this genus contain a toxic amino-acid which can cause 'lathyrism' (a disease of the nervous system) if ingested in large quantities. The amino-acid is produced at about the same time that the seed starts to develop.
					(Lathyrus sylvestris)	See ***Roviglie***				
Mochi	Seeds	The seeds are boiled to make a mush to be mixed with the bread dough			*Lathyrus cicera* L.	Fabaceae	Yes			All parts of the plant, but especially the seed, contain a toxic amino-acid which can cause 'lathyrism' (a disease of the nervous system) if ingested in large quantities. The amino-acid is produced at about the same time that the seed starts to develop.
					(Ervilia sativa)	See ***Veggioli***				

More di Rovo	Fruits	Small fruits, dried, can be added directly to bread dough			*Rubus caesius* L.	Rosaceae	Yes			
Moro	Tips or tops and tender twigs	Tips, tops or tender twigs must be cooked before adding them to bread dough			*Morus alba* L.	Moraceae	Yes			
					Morus nigra		No			
Mortella	Fruits	Sour fruits, fresh or dried, are cooked in water, sieved and then added to bread dough			*Myrtus communis* L.	Myrtaceae	Yes			
Mustin Greco salvatico detto Gettaione	Roots	The roots, boiled a first time with a small bag of ashes and a second time with pure water, are sieved and added to the bread dough			*Agrostemma githago* L.	Caryophyllaceae	Yes	T	T	
Narcisi salvatichi detti Moschettoni	Roots	The roots, boiled a first time with a small bag of ashes and a second time with pure water, are sieved and added to the bread dough			*Narcissus pseudonarcissus* L.	Amaryllidaceae	No		T	
Nasturzio Aquatico<br><br>***Sisimbrio Aquatico***	Leaves	Leaves are useful against scurvy			*Rorippa palustris* (L.) Besser	Brassicaceae	No			
Nepitella	Leaves and tops	Dried and pulverized leaves and tops, can be added to bread dough to improve the flavour of bland, cloying or flatulent mixtures			*Clinopodium nepeta* (L.) Kuntze subsp. *nepeta*	Lamiaceae	Yes			
Nespole salvatiche	Fruits	Sour fruits, fresh or dried, are cooked in water, sieved and then added to bread dough		C	*Mespilus germanica* L.	Rosaceae	Yes			
Ninfea di fior giallo	Seeds<br><br>Roots	Seeds crushed and pressed in water produce an aromatic water that serves as a flavouring agent for baking flours with a sour or bitter taste<br>Young roots, fresh or boiled, are crushed and added to bread dough			*Nuphar lutea* (L.) Sm.	Nymphaeaceae	Yes			
Ninfea fiori bianchi	Seeds<br><br>Roots	Seeds crushed and pressed in water produce an aromatic water that serves as a flavouring agent for baking flours with a sour or bitter taste<br>Young roots, fresh or boiled, are crushed and added to bread dough			*Nymphaea alba* L.		Yes		T	The toxic alkaloids *nupharine* and *nymphaeine* have an effect on the nervous system
Nocciuole o Avellane salvatiche	Seeds	Dried seeds, once peeled, are ground in order to obtain flour to be mixed with bread dough			*Corylus avellana* L.	Betulaceae	Yes			
Noci	Seeds	The seeds can be used to modify the bitter and sour taste of some acorns			*Juglans regia* L.	Juglandaceae	Yes			
Nummularia	Tips or tops and tender twigs	Tips, tops or tender twigs must be cooked before adding them to bread dough			*Lysimachia nummularia* L.	Primulaceae	No			

Ocimoide bianco	Tips or tops and tender twigs	Tips, tops or tender twigs must be cooked before adding them to bread dough			(*Silene dioica* L.)	See ***Linnide***				
					Silene latifolia Poir.	Caryophyllaceae	Yes			The plant contain saponins, which are very poorly absorbed by the body and are also broken down by thorough cooking
Ocro	Seeds	The seeds are boiled to make a mush to be mixed with the bread dough			*Lathyrus ochrus* (L.) DC.	Fabaceae	Yes			Seeds might be poisonous in large quantities
Onobrichide	Leaves and tops	Raw leaves and tops are crushed and added to bread dough			*Onobrychis viciifolia* Scop.		Yes			
Onopordo o Acanzio	Tips or tops and tender twigs	Tips, tops or tender twigs must be cooked before adding them to bread dough			*Onopordum acanthium* L.	Asteraceae	Yes			
Origano detto Regamo ed Erba da Acciughe	Leaves and tops	Dried and pulverized leaves and tops, can be added to bread dough to improve the flavour of bland, cloying or flatulent mixtures			*Origanum vulgare* L.	Lamiaceae				
Ornitogalo bianco o Latte di gallina	Roots	The roots, boiled a first time with a small bag of ashes and a second time with pure water, are sieved and added to the bread dough			*Ornithogalum umbellatum* L.	Asparagaceae	Yes		T	The bulb contains alkaloids and is poisonous.
Ornitogalo giallo	Roots	The roots, boiled a first time with a small bag of ashes and a second time with pure water, are sieved and added to the bread dough			*Gagea lutea* (L.) Ker Gawl.	Liliaceae	No			
Ortica	Young sprouts	Cooked young sprouts can be added to bread, but it would be better to eat them alone			*Urtica dioica* L.	Urticaceae	Yes			The stinging hairs are neutralized by heat or by thorough drying. But only young leaves should be used because older leaves develop gritty particles called *cystoliths* which act as an irritant to the kidneys.
					Urtica urens L.		Yes			
Orzo, Orzuola	Seeds	Dried seeds are ground into bread flour		C	*Hordeum vulgare* L.	Poaceae	No			Possible trigger for coeliac disease.
Ossiacanta o Marruca bianca	Fruits	Sour fruits, fresh or dried, are cooked in water, sieved and then added to bread dough			*Crataegus laevigata* (Poir.) DC.	Rosaceae	Yes			
Pancaciuolo detto Spadicciuola	Roots	The roots, boiled to one water and sieved can be added to the bread dough			*Gladiolus communis* L.	Iridaceae	Yes			
					Bunium bulbocastanum	Apiaceae	Yes			
Parietaria o Vetriuola	Leaves	Leaves are boiled, if necessary twice with water change, then pounded, sieved and added to bread dough			*Parietaria officinalis* L.	Urticaceae	Yes			
Pastinache	Roots	Roots, dried in pieces, can be crushed and mixed to bread dough. Alternatively, fresh pieces can be cooked, sieved and then added to bread dough		C	*Pastinaca sativa* L. subsp. *sativa*	Apiaceae	Yes			It is said to contain myristicine, which is toxic in excess
				C	*Pastinaca sativa* L. subsp. *urens* (Req. ex Godr.) Čelak.		Yes			

Pentafillo detto Cinque Foglie	Tips or tops and tender twigs	Tips, tops or tender twigs must be cooked before adding them to bread dough			*Potentilla reptans* L.	Rosaceae	Yes			
Peonia	Roots	The procedure is described in table 1	T		*Paeonia officinalis* L.	Paeoniaceae	No	T		It is poisonous if taken in large doses
Pere<br>***Pero***	Fruits<br>Tips or tops and tender twigs	Fruits, dried in segments, can be crushed and mixed to bread dough; alternatively, fresh or dried segments can be cooked first and then added to bread dough<br>Tips, tops or tender twigs must be cooked before adding them to bread dough		C	*Pyrus communis* L.	Rosaceae	Yes			
Perecotogne	Fruits	Sour fruits, fresh or dried, are cooked in water, sieved and then added to bread dough		C	*Cydonia oblonga* Mill.		Yes			
Pesche	Fruits	Fruits, dried in segments, can be crushed and mixed to bread dough; alternatively fresh or dried segments can be cooked first and then added to bread dough		C	*Prunus persica* (L.) Batsch		Yes			
Petasite	Roots	The roots, boiled a first time with a small bag of ashes and a second time with pure water, are sieved and added to the bread dough			*Petasites albus* (L.) Gaertn.	Asteraceae	Yes	T		
					Petasites hybridus (L.) G. Gaertn., B. Mey. & Scherb.		Yes	T		
Piantaggine, Quinquenervia	Leaves	Leaves are boiled, if necessary twice with water change, then pounded, sieved and added to bread dough			*Plantago lanceolata* L.	Plantaginaceae	Yes	T		
					Plantago major L.		Yes			High doses may cause a fall in blood pressure and diarrhoea. Possible allergic contact dermatitis. Avoid in patients with intestinal obstruction or abdominal discomfort
					Plantago media L.		Yes			
Pimpinella detta Salvastrella	Tips or tops and tender twigs	Tips, tops or tender twigs must be cooked before adding them to bread dough			*Poterium sanguisorba* L.	Rosaceae	Yes			
Pinocchi	Seeds	Dried seeds are ground in order to obtain flour to be mixed with bread dough			*Pinus pinea* L.	Pinaceae	Yes			
Piracanta o Pruno Gazzerino	Fruits	Sour fruits, fresh or dried, are cooked in water, sieved and then added to bread dough			*Pyracantha coccinea* M. Roem.	Rosaceae	No			
Piselli	Seeds<br>Leaves and tops	The seeds are boiled to make a mush to be mixed with the bread dough<br>Raw leaves and tops are crushed and added to bread dough		C	*Lathyrus oleraceus* Lam.	Fabaceae	Yes			
Poligonato o Sigillo di Salomone	Young sprouts	Cooked young sprouts can be added to bread, but it would be better to eat them alone			*Polygonatum multiflorum* (L.) All.	Asparagaceae	Yes		T	

Polmonaria	Leaves and tops	Raw leaves and tops are crushed and added to bread dough			*Pulmonaria officinalis* L.	Boraginaceae	Yes			
Pomi di Terra	Roots	There is only one indication for a desirable, but not yet possible use		C	*Solanum tuberosum* L.	Solanaceae	No	T		
Poponi, Zatte	Fruits<br><br>Tender shoots and vines	Boiled fruit pulp and peel is added to bread dough<br>Cooked tender shoots and vines can be added to bread, but it would be better to eat them alone		C	*Cucumis melo* L.	Cucurbitaceae	No			
Populus alba o Gattice	Tips or tops and tender twigs	Tips, tops or tender twigs must be cooked before adding them to bread dough			*Populus alba* L.		No			
Populus nigra detta Albero	Tips or tops and tender twigs	Tips, tops or tender twigs must be cooked before adding them to bread dough			*Populus nigra* L.	Salicaceae	No			
Populus tremula	Tips or tops and tender twigs	Tips, tops or tender twigs must be cooked before adding them to bread dough			*Populus tremula* L.		No			
Porraccio	Roots	The procedure is described in Table 1	T		*Asphodelus albus* Mill.	Asphodelaceae	Yes			The roots are poisonous
Porreca	Roots	The procedure is described in Table 1	T		*Asphodelus fistulosus* L.		Yes			
Portulaca o Porcellana	Leaves and tops	Raw leaves and tops are crushed and added to bread dough			*Portulaca oleracea* L.	Portulacaceae	Yes			
Potamogeto con foglie di Piantaggine	Leaves and tops	Raw leaves and tops are crushed and added to bread dough			*Potamogeton natans* L.	Potamogetonaceae	Yes			
Potentilla o Argentina	Leaves	Leaves are boiled, if necessary twice with water change, then pounded, sieved and added to bread dough			*Argentina anserina* (L.) Rydb.	Rosaceae	Yes			Possible stomach irritation
Prezzemolo	Leaves and Seeds	Dried and pulverized leaves and seeds, can be added to bread dough to improve the flavour of bland, cloying or flatulent mixtures		C	*Petroselinum crispum* (Mill.) Fuss	Apiaceae	Yes	T		It is said to contain myristicine, which is toxic in excess
Primulaveris	Leaves and tops	Raw leaves and tops are crushed and added to bread dough			*Primula veris* L.	Primulaceae	Yes			Saponins may cause hypotension.
Pseudoacoro o Iride palustre dal fior giallo	Roots	The procedure is described in table 1	T		*Limniris pseudacorus* (L.) Fuss	Iridaceae	No		T	The roots are especially likely to be toxic. Should not be used internally except in small doses.
Puleggio	Leaves and tops	Dried and pulverized leaves and tops, can be added to bread dough to improve the flavour of bland, cloying or flatulent mixtures			*Mentha pulegium* L.	Lamiaceae	Yes			In large quantities it can cause abortions

Querce	Seeds<br>Tips or tops and tender twigs	Dried seeds are ground in order to obtain flour. Before using it, acorn flour must be boiled until it becomes a liquid paste, then it is added to bread dough<br>Tips, tops or tender twigs must be cooked before adding them to bread dough			*Quercus petraea* (Matt.) Liebl.	Fagaceae	Yes			
					Quercus pubescens Willd.		Yes			
Radicchio salvatico	Roots<br>Leaves	The roots, boiled a first time with a small bag of ashes and a second time with pure water, are sieved and added to the bread dough<br>Leaves are boiled, if necessary twice with water change, then pounded, sieved and added to bread dough			*Cichorium intybus* L.	Asteraceae	Yes			Excessive and continued use may impair function of the retina.
					Crepis zacintha (L.) Loisel.		Yes			
Ramerino	Leaves and tops	Boil leaves and tops with legumes, or use their decoction to knead bread that contains legume flour, to avoid mixtures that may cause flatulence<br>The leaves and tops can be used to modify the bitter and sour taste of some acorns<br>Dried and pulverized leaves and tops, can be added to bread dough to improve the flavour of bland, cloying or flatulent mixtures			*Salvia rosmarinus* Spenn.	Lamiaceae	Yes			In large quantities it can cause abortions
Ramolacci	Roots	Roots, dried in pieces, can be crushed and mixed to bread dough. Alternatively, fresh pieces can be cooked, sieved and then added to bread dough			*Raphanus raphanistrum* L.	Brassicaceae	Yes			
Rape	Roots<br>Leaves	Roots, dried in pieces, can be crushed and mixed to bread dough. Alternatively, fresh pieces can be cooked, sieved and then added to bread dough<br>Leaves are boiled, if necessary twice with water change, then pounded, sieved and added to bread dough		C	*Brassica rapa* L.		Yes			
Raperonzoli	Roots	Roots, dried in pieces, can be crushed and mixed to bread dough. Alternatively, fresh pieces can be cooked, sieved and then added to bread dough			*Campanula rapunculus* L.	Campanulaceae	Yes			
					Tragopogon porrifolius L.	Asteraceae	Yes			
Riso	Seeds	Dried seeds are ground into bread flour		C	*Oryza sativa* L.	Poaceae	Yes			
Robbia	Young sprouts	Cooked young sprouts can be added to bread, but it would be better to eat them alone			*Galium mollugo* L.	Rubiaceae	Yes			
Romice	Roots	The dried roots are crushed and sieved and the resulting powder is boiled, then decanted and the water is thrown away to add new water and boiled a second time, then the powder is added to the bread dough			*Rumex crispus* L.	Polygonaceae	Yes			It can contain high levels of oxalic acid, the quantity of which can be reduced by cooking.
					Rumex pulcher L.		Yes			
Rose salvatiche					*Rosa agrestis* Savi	Rosaceae	Yes			

	Tips or tops and tender twigs	Tips, tops or tender twigs must be cooked before adding them to bread dough			*Rosa arvensis* Huds.		Yes			
					Rosa canina L.		Yes			If ingested, the layer of hairs around the seeds just beneath the flesh of the fruit can cause irritation to the mouth and digestive tract
					Rosa gallica L.		Yes			
					Rosa sempervirens L.		Yes			
					Rosa spinosissima L.		No			
Rosoni	Roots	The roots, boiled a first time with a small bag of ashes and a second time with pure water, are sieved and added to the bread dough			*Alcea rosea* L.	Malvaceae	Yes			
Rovi	Tips or tops and tender twigs	Tips, tops or tender twigs must be cooked before adding them to bread dough			*Rubus ulmifolius* L.	Rosaceae	Yes			
Roviglie	Seeds<br><br>Leaves and tops	The seeds are boiled to make a mush to be mixed with the bread dough<br>Raw leaves and tops are crushed and added to bread dough			*Lathyrus sylvestris* L.	Fabaceae	Yes		T	All parts of the plant, but especially the seed, contain a toxic amino-acid which can cause 'lathyrism' (a disease of the nervous system) if ingested in large quantities. The amino-acid is produced at about the same time that the seed starts to develop.
Ruchetta	Leaves<br><br>Roots	Leaves are useful against scurvy			*Diplotaxis muralis* (L.) DC.	Brassicaceae	Yes			
					Eruca vesicaria (L.) Cav.		Yes			
Rusco detto Pungitopo	Young sprouts	Cooked young sprouts can be added to bread, but it would be better to eat them alone			*Ruscus aculeatus* L.	Asparagaceae	Yes			
Saggina	Seeds	Dried seeds are ground into bread flour		C	*Sorghum bicolor* (L.) Moench subsp. *bicolor*	Poaceae	No	T		
Salicornia detta Salsuola	Leaves and tops	Finely crushed and sifted, are used instead of salt to flavour bread doughs			*Salicornia fruticosa* (L.) L.	Amaranthaceae	Yes			
					Salicornia perennans Willd.		Yes			
Salvia	Leaves<br><br>Leaves and tops	The leaves can be used to modify the bitter and sour taste of some acorns<br>Dried and pulverized leaves and tops, can be added to bread dough to improve the flavour of bland, cloying or flatulent mixtures			*Salvia officinalis* L.	Lamiaceae	Yes			It can be toxic when used in excess or when taken for extended periods.
Sambuco	Flowers	Dried and pulverized flowers, can be added to bread dough to improve the flavour of bland, cloying or flatulent mixtures			*Sambucus nigra* L.	Adoxaceae	Yes	T		
Sassefrica	Leaves and tops	Raw leaves and tops are crushed and added to bread dough			*Tragopogon pratensis* L.	Asteraceae	Yes			
					Satureja hortensis L.	Lamiaceae	Yes			

Satureia detta Santoreggia	Leaves and tops	Boil Leaves and tops with legumes, or use their decoction to knead bread that contains legume flour, to avoid mixtures that may cause flatulence<br>Dried and pulverized leaves and tops, can be added to bread dough to improve the flavour of bland, cloying or flatulent mixtures			*Satureja montana* L.		Yes			
Scabiosa	Tips or tops and tender twigs	Tips, tops or tender twigs must be cooked before adding them to bread dough			*Stachys arvensis* (L.) L.		No			
Sclarea detta Scarleggia	Leaves and tops	Dried and pulverized leaves and tops, can be added to bread dough to improve the flavour of bland, cloying or flatulent mixtures			*Salvia sclarea* L.		Yes			
Scolimo	Young sprouts	Cooked young sprouts can be added to bread, but it would be better to eat them alone			*Cynara cardunculus* subsp. *scolymus (L.) Hegi*	Asteraceae	Yes			
Scorzonere, Scorzonera	Roots<br><br>Leaves and tops	Roots, dried in pieces, can be crushed and mixed to bread dough. Alternatively, fresh pieces can be cooked, sieved and then added to bread dough<br>Raw leaves and tops are crushed and added to bread dough			*Pseudopodospermum hispanicum* (L.) Zaika, Sukhor. & N. Kilian		Yes			
					Scorzonera humilis L.		No			
Sedi o Semprevivi	Leaves and tops	Raw leaves and tops are crushed and added to bread dough			*Sedum album* L.	Crassulaceae	Yes		T	
					Sedum cepaea L.		Yes			
					Sempervivum tectorum L.		Yes			
Segale	Seeds	Dried seeds are ground into bread flour		C	*Secale cereale* L.	Poaceae	No			
Senapa domestica	Leaves	Leaves are useful against scurvy		C	*Brassica nigra* (L.) W.D.J. Koch	Brassicaceae	Yes			Mustard allergy possibly especially in children and adolescents. Retention of seeds possibly in intestines if taken internally.
					Sinapis alba L.		Yes			It is possibly poisonous once the seedpods have formed.
					Sinapis arvensis L.		Yes			
Senapa salvatica	Leaves	Leaves are useful against scurvy			*Sisymbrium irio* L.		Yes			
					Sisymbrium officinale (L.) Scop.		Yes			
Senecione o Erba Calderugia	Leaves and tops	Raw leaves and tops are crushed and added to bread dough			*Senecio vulgaris* L.	Asteraceae	Yes	T	T	Presence of a liver-affecting toxin.
Sermollino Cedrato	Leaves and tops	Dried and pulverized leaves and tops, can be added to bread dough to improve the flavour of bland, cloying or flatulent mixtures			*Thymus longicaulis* C.Presl.	Lamiaceae	Yes			
					Thymus praecox Opiz		Yes			
					Thymus pulegioides L.		Yes			

Serpillo detto Sermollino	Leaves and tops	Dried and pulverized leaves and tops, can be added to bread dough to improve the flavour of bland, cloying or flatulent mixtures			*Thymus vulgaris* L.		Yes			Avoid if inflammatory disease of the gastrointestinal tract. Internal use contraindicated especially in pregnancy. Caution if sensitive to grasses
Sinfito o Consolida maggiore	Roots<br><br>Leaves and tops	The roots, boiled and sieved can be added to the bread dough<br>Raw leaves and tops are crushed and added to bread dough			*Symphytum officinale* L.	Boraginaceae	Yes	T		Presence of a liver-affecting toxic alkaloid in the roots (large quantities) and leaves (higher as they grow older, almost none in young leaves).
Sinfito di radica tuberosa	Roots	The roots, boiled and sieved can be added to the bread dough			*Symphytum tuberosum* subsp. *angustifolium* (A. Kern.) Nyman		Yes	T		Possible presence of a liver-affecting toxic alkaloid.
Sio Montano	Leaves and Seeds	Dried and pulverized leaves and seeds, can be added to bread dough to improve the flavour of bland, cloying or flatulent mixtures			*Sium latifolium* L.	Apiaceae	No			The entire plant, and especially the root, is poisonous
Sio Palustre detto Crescione	Leaves and Seeds	Dried and pulverized leaves and seeds, can be added to bread dough to improve the flavour of bland, cloying or flatulent mixtures			*Helosciadium nodiflorum* (L.) W.D.J. Koch		Yes			
Sisimbrio Aquatico	Leaves	Leaves are useful against scurvy			*Nasturtium officinale* W.T. Aiton	Brassicaceae	Yes			Risk of it being infested with the liver fluke parasite; cooking the leaves will destroy any parasites. May inhibit the metabolism of paracetamol.
Solidaggine Sarracenica	Tips or tops and tender twigs	Tips, tops or tender twigs must be cooked before adding them to bread dough			*Senecio ovatus* (G. Gaertn., B. Mey. & Scherb.) Willd.	Asteraceae	No	T	T	
Sorbo aucupario detto Sorbezzolo*	Fruits	Sour fruits, fresh or dried, are cooked in water, sieved and then added to bread dough			*Sorbus aucuparia* L.	Rosaceae	Yes			Large quantities of the raw fruit can cause vomiting.
Sorbo Torminale	Fruits	Sour fruits, fresh or dried, are cooked in water, sieved and then added to bread dough			*Sorbus torminalis* (L.) Crantz		Yes			
Sparagi salvatichi<br><br>***Sparagio Salvatico***	Roots,<br><br>Young sprouts	The roots, boiled a first time with a small bag of ashes and a second time with pure water, are sieved and added to the bread dough<br>Cooked young sprouts can be added to bread, but it would be better to eat them alone			*Asparagus acutifolius* L.	Asparagaceae	Yes			
Spinaci	Leaves			C	*Spinacia oleracea L.*	Amaranthaceae	No			Leaves contain oxalic acid, the quantity of which can be reduced by cooking.
Spinacione<br><br>***Spinacioni***	Leaves and tops	Raw leaves and tops are crushed and added to bread dough		C	*Atriplex hortensis* L.		Yes			Leaves can concentrate harmful amounts of nitrates if the plant grows on nitrogen-rich soils<br><br>The seed contains *saponins* that can be removed by carefully leaching the seed or flour in running water or thorough cooking (and perhaps changing the cooking water once).

Sughero	Seeds	Dried seeds are ground in order to obtain flour. Before using it, acorn flour must be boiled until it becomes a liquid paste, then it is added to bread dough			*Quercus suber* L.	Fagaceae	Yes			
Susine domestiche	Fruits	Fruits, dried in segments, can be crushed and mixed to bread dough; alternatively fresh or dried segments can be cooked first and then added to bread dough		C	*Prunus domestica* L.	Rosaceae	Yes			
Susine salvatiche o prugnole o di macchia	Fruits	Sour fruits, fresh or dried, are cooked in water, sieved and then added to bread dough			*Prunus spinosa* L. subsp. *spinosa*		Yes			
Tamerigia	Tips or tops and tender twigs	Tips, tops or tender twigs must be cooked before adding them to bread dough			*Tamarix gallica* L.	Tamaricaceae	No			
Terracrepolo	Leaves and tops	Raw leaves and tops are crushed and added to bread dough			*Reichardia picroides* (L.) Roth	Asteraceae	Yes			
Tino detto Lentaggine	Tips or tops and tender twigs	Tips, tops or tender twigs must be cooked before adding them to bread dough			*Viburnum tinus* L.	Adoxaceae	No		T	
Tormentilla	Roots<br><br>Tips or tops and tender twigs	The dried roots are crushed and sieved and the resulting powder is boiled, then decanted and the water is thrown away to add new water and boiled a second time, then the powder is added to the bread dough<br>Tips, tops or tender twigs must be cooked before adding them to bread dough			*Potentilla erecta* (L.) Raeusch.	Rosaceae	No			Gastrointestinal symptoms if doses over 1g.
Tosillaggine o Farfero	Roots<br><br>Leaves	The roots, boiled to one water and sieved can be added to the bread dough<br>Leaves are boiled, if necessary twice with water change, then pounded, sieved and added to bread dough			*Tussilago farfara* L.	Asteraceae	Yes	T		It contains liver-affecting pyrrolizidine alkaloids (potentially toxic in large doses).
Tribolo acquatico	Seeds	Dried seeds are ground in order to obtain flour to be mixed with bread dough			*Trapa natans* L.	Lythraceae	Yes			Raw seeds contain toxins that are destroyed by cooking.
Trifoglio Acetoso	Leaves	The leaves can be used to give a pretty sour flavour to unflavoured or too sweet bread doughs; they are useful against scurvy			*Oxalis acetosella* L.	Oxalidaceae	Yes	T		Leaves contain oxalic acid, the quantity of which can be reduced by cooking.
Tulipano giallo salvatico	Roots	The roots, boiled a first time with a small bag of ashes and a second time with pure water, are sieved and added to the bread dough			*Tulipa sylvestris* L.	Liliaceae	Yes		T	Bulbs and flowers can cause dermatitis in sensitive people
Valeriana rossa o pisana	Leaves and tops	Raw leaves and tops are crushed and added to bread dough			*Centranthus ruber* (L.) DC.	Caprifoliaceae	Yes			
Vecce	Seeds	The seed is dried and ground into flour			*Lathyrus hirsutus* L.	Fabaceae	No			

					Lathyrus pratensis L.		Yes			The seed might be poisonous in large quantities
					Vicia lathyroides L.		No			
					Vicia monantha Retz. subsp. *calcarata* (Desf.) Romero Zarco		No			
Vecce domestiche	Tips or tops and tender twigs	Tips, tops or tender twigs must be cooked before adding them to bread dough		C	*Vicia sativa* L.		Yes			The seed might be poisonous in large quantities
Vecce Salvatiche	Seeds<br><br>Tips or tops and tender twigs	The seeds are boiled to make a mush to be mixed with the bread dough<br>Tips, tops or tender twigs must be cooked before adding them to bread dough			*Vicia lutea* L.		No			
					Vicia sepium L.		No			
Veggioli	Seeds	The seeds are boiled to make a mush to be mixed with the bread dough			*Ervilia sativa* Link		Yes			The seed might be poisonous in large quantities
Vena	Seeds	Dried seeds are ground into bread flour		C	*Avena sativa* L.	Poaceae	No			
Verbena	Tips or tops and tender twigs	Tips, tops or tender twigs must be cooked before adding them to bread dough			*Verbena officinalis* L.	Verbenaceae	Yes	T		Verbenalin: can cause nervous system paralysis resulting in stupor and convulsion.<br>Allopathic medicine for high blood pressure can be effected by vervain. Uterus stimulation possible – avoid during pregnancy. Avoid if breast feeding. If allergies or fits use with caution
Veronica maschia	Tips or tops and tender twigs	Tips, tops or tender twigs must be cooked before adding them to bread dough			*Veronica chamaedrys* L.	Plantaginaceae	Yes			
					Veronica officinalis L.		No			
Viole mammole	Leaves	Leaves are boiled, if necessary twice with water change, then pounded, sieved and added to bread dough			*Viola odorata* L.	Violaceae	Yes			May be cause vomiting; possible additive effect with laxatives
Virga Aurea	Tips or tops and tender twigs	Tips, tops or tender twigs must be cooked before adding them to bread dough			*Solidago virgaurea* L.	Asteraceae	No			Mild allergic reactions. Avoid during pregnancy and breast feeding. Care if chronic kidney disease.
Vite domestica	Fruits<br><br>Tender shoots and vines<br><br>Tendrils	Small fruits, dried, can be added directly to bread dough; dried and crushed pomace can be added to flour with a bitter taste<br>Cooked tender shoots and vines can be added to bread, but it would be better to eat them alone<br>The young tendrils can be used to give a pretty sour flavour to unflavoured or too sweet bread doughs; they are useful against scurvy		C	*Vitis vinifera* L.	Vitaceae	Yes			

Vulneraria	Tips or tops and tender twigs	Tips, tops or tender twigs must be cooked before adding them to bread dough			*Anthyllis vulneraria* L.	Fabaceae	No			
Zucche	Fruits<br>Tender shoots and vines	Boiled fruit pulp is added to bread dough<br>Cooked tender shoots and vines can be added to bread, but it would be better to eat them alone		C	*Cucurbita maxima* Duchesne	Cucurbitaceae	No			
				C	*Cucurbita pepo* L.		No			

Table S2. List of vernacular names used by Giovanni Targioni Tozzetti [6] for which a match could not be found. Column headings as in Table S1.

Vernacular name	PU	Procedure	Species
Medicaggine	Leaves and tops	Raw leaves and tops are crushed and added to bread dough	-
Cipperoidi	Seeds	Dried seeds are ground in order to obtain flour to be mixed with bread dough	-
Giunchi	Seeds<br>Roots	Dried seeds are ground in order to obtain flour to be mixed with bread dough<br>Young roots, fresh or boiled, are crushed and added to bread dough	*Juncus* sp. pl., *Scirpus* sp. pl., other species
Latiro	Seeds	The seeds are boiled to make a mush to be mixed with the bread dough	*Lathyrus* sp.
Rose domestiche	Tips or tops and tender twigs	Tips, tops or tender twigs must be cooked before adding them to bread dough	*Rosa* sp. pl.
Salci	Tips or tops and tender twigs	Tips, tops or tender twigs must be cooked before adding them to bread dough	*Salix* sp. pl.
Trifoglio	Leaves and tops	Raw leaves and tops are crushed and added to bread dough	*Lotus* sp. pl., *Dorycnium* sp. pl., *Trifolium* sp. pl.

Table S3. Section A): Taxa in common (37) between list of species from the AlimurgITA database with the one proposed by Giovanni Targioni Tozzetti [6]; Section B): taxa NOT in common (16) between the list of species from the AlimurgITA database with the one proposed by Giovanni Targioni Tozzetti [6].

**Species**	**Use**	**Used part**
Section A		
Artemisia vulgaris L.	bread flavoring	leaves
Arum italicum Mill. subsp. *italicum*	production of flour	roots
Asphodelus albus Mill. subsp. *subalpinus* Nyman	production of flour	roots
Bistorta officinalis Delarbre	production of flour	roots
Castanea sativa Mill.	production of flour	fruits
Chenopodium album L.	production of flour	seeds
Cyclamen hederifolium Aiton	production of flour	roots
Cyclamen repandum Sm. subsp. *repandum*	production of flour	roots
Elymus repens (L.) Gould subsp. *repens*	production of flour, bread flavoring	roots, fruits
Ervilia sativa Link	production of flour	seeds
Fagopyrum esculentum Moench	production of flour	seeds
Ficaria verna Huds.	production of flour	roots
Ficus carica L.	bread flavoring	fruits
Foeniculum vulgare Mill.	bread flavoring	fruits
Galium aparine L.	production of flour	seeds
Humulus lupulus L.	bread flavoring	flowers
Juglans regia L.	bread flavoring	seeds
Lathyrus sylvestris L. subsp. *sylvestris*	production of flour	seeds
Linum usitatissimum L.	production of flour	seeds
Lotus tetragonolobus L.	production of flour	seeds
Malva sylvestris L.	bread flavoring	seeds
Medicago sativa L.	production of flour	seeds
Nymphaea alba L.	production of flour	seeds, roots
Origanum vulgare L. (incl. subsp. *viridulum* (Martrin-Donos) Nyman	bread flavoring	leaves, flowers
Oryza sativa L. subsp. *sativa*	production of flour	seeds
Panicum miliaceum L.	production of flour	seeds

Pistacia lentiscus L.	bread flavoring	seeds
Pyrus communis L.	bread flavoring	fruits
Quercus ilex L. subsp. *ilex*	production of flour	fruits
Quercus pubescens Willd. subsp. *pubescens*	production of flour	fruits
Quercus robur L.	production of flour	fruits
Quercus suber L.	production of flour	fruits
Raphanus raphanistrum L.	bread flavoring	leaves
Rosa canina L.	production of flour	fruits
Salvia rosmarinus Spenn.	bread flavoring	leaves
Schoenoplectus lacustris (L.) Palla	production of flour	roots
Vicia sativa L.	production of flour	seeds
Section B		
Allium subhirsutum L. subsp. *subhirsutum*	bread flavoring	roots
Bassia scoparia (L.) Voss	production of flour	seeds
Betula pendula Roth	production of flour	barks
Betula pubescens Ehrh.	production of flour	barks
Blitum bonus-henricus (L.) Rchb.	production of flour	seeds
Brachypodium sylvaticum (Huds.) P.Beauv. subsp. *sylvaticum*	production of flour	roots
Carum carvi L.	bread flavoring	fruits
Ceratonia siliqua L.	production of flour	seeds
Erythronium dens-canis L.	production of flour	roots
Galium aparine L.	production of flour	seeds
Lolium pratense (Huds.) Darbysh.	production of flour	seeds
Loncomelos pyrenaicum (L.) L.D.Hrouda	bread flavoring	seeds
Malva parviflora L.	bread flavoring	seeds
Nigella arvensis L.	bread flavoring	fruits
Sorbus aria (L.) Crantz	production of flour	fruits
Thymbra capitata (L.) Cav.	bread flavoring	leaves
